# Cancer progression modeling using static sample data

**DOI:** 10.1186/s13059-014-0440-0

**Published:** 2014-08-26

**Authors:** Yijun Sun, Jin Yao, Norma J Nowak, Steve Goodison

**Affiliations:** Department of Microbiology and Immunology, Department of Computer Science and Engineering, Department of Biostatistics, University at Buffalo, The State University of New York, Buffalo, 14201 NY USA; Department of Bioinformatics and Biostatistics, Roswell Park Cancer Institute, Buffalo, 14201 NY USA; Department of Electrical and Computer Engineering, University of Florida, Gainesville, 32610 FL USA; Department of Biochemistry, University at Buffalo, The State University of New York, Buffalo, 14201 NY USA; Department of Health Sciences Research, Mayo Clinic, Jacksonville, 32224 FL USA

## Abstract

As molecular profiling data continue to accumulate, the design of integrative computational analyses that can provide insights into the dynamic aspects of cancer progression becomes feasible. Here, we present a novel computational method for the construction of cancer progression models based on the analysis of static tumor samples. We demonstrate the reliability of the method with simulated data, and describe the application to breast cancer data. Our findings support a linear, branching model for breast cancer progression. An interactive model facilitates the identification of key molecular events in the advance of disease to malignancy.

**Electronic supplementary material:**

The online version of this article (doi:10.1186/s13059-014-0440-0) contains supplementary material, which is available to authorized users.

## Background

Human cancer is a dynamic disease that develops over an extended time period through the accumulation of a series of genetic alterations. Once initiated, the advance to malignancy can to some extent be considered a Darwinian process (a multistep evolutionary process) that responds to selective pressure [1-5]. While the majority of genetic alterations confer no growth advantage, tumor cells that acquire mutations in genes that control key cellular processes can overwhelm less vigorous cell populations within the tumor mass, and this process continues through a series of clonal expansions that result in tumor persistence and growth, and ultimately the ability to invade surrounding tissues and metastasize to distant organs. The delineation of this dynamic process and the identification of pivotal molecular events that drive stepwise progression to malignancy would provide a critical foundation and guide for the development of cancer diagnostics, prognostics and targeted therapeutics.

The assembly of time-series data collected through repeated sampling across an entire disease process would provide essential information for the elucidation of system dynamics and disease-associated genetic regulation [6], and recent advances in genomic technologies has made it possible to study cancer genomes at a scale and cost that allow the design of such studies [7,8]. However, due to the need for immediate treatment upon diagnosis, it is ethically infeasible to collect time-series data to study disease progression. An alternative is animal studies where time-series data could be obtained, but the extrapolation of animal data to human disease is not always appropriate. Moreover, the development of spontaneous human cancer is a process that takes several years, and to overcome the heterogeneous nature of the disease and to minimize random effects, a very large number of samples is required, so animal studies are not economically or logistically viable in this context. Conversely, due to the high incident rate of cancer, and the clinical care protocols in place in developed countries, a huge number of archived tumor specimens is available. For example, it is estimated that about 250,000 new cases of breast cancer will be diagnosed in 2014 in the US [9]. Assuming that cancer cells are derived from normal cells, and that a static sample can be regarded as a snapshot of the dynamic cancer process, then we can pose the following question: *Is it possible to construct a cancer progression model using data acquired from static samples?*

**Figure 1:**
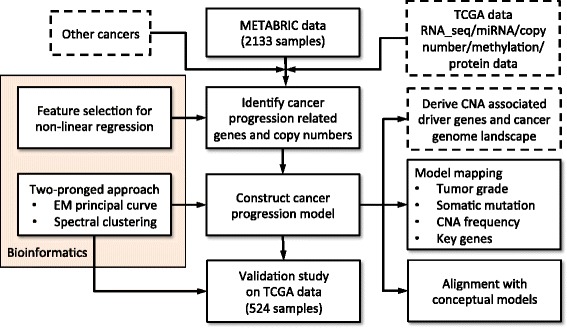
**Workflow of the stepwise study.** The tasks listed in the dashed-line rectangles are possible extensions and applications of the study and are discussed in the text. CNA, copy number alteration; EM, expectation-maximization; miRNA, microRNA; RNA_seq, RNA sequencing; TCGA, The Cancer Genome Atlas.

In this paper, we present a proof-of-principle population study to address the above question. Figure 1 depicts a flowchart of the presented stepwise study. It involves extensive work on algorithm development, computational simulation, disease model construction and validation. By analysis of publicly available data, we demonstrated that through the application of advanced computational techniques it is *indeed* possible to construct a cancer progression path using massive data obtained from static sampling. Analysis of molecular data from 2,133 breast samples [7] enabled the visualization of high-dimensional data structures that provided a framework for the extrapolation of progressive disease associations and trends across tumor samples. A very similar data structure was revealed through the analysis of an independent dataset of 524 samples [8], and mapping of copy number alteration (CNA) frequencies, somatic mutation rates, tumor grade information, and the expression levels of specific key genes supported the validity of the model. Our analysis, *for the first time*, showed that while breast cancer is a genetically and clinically heterogeneous disease, tumor samples are distributed on a low-dimensional data structure manifold, suggesting that genotypes are not hard-wired and can shift over time.

The study provides a framework for the construction of high-resolution cancer progression models that can combine all currently available genetic information. Through the visualization of key events in tumor progression, such models can facilitate the identification of pivotal driver genes, improved prognostic stratification systems and potential points of susceptibility for therapeutic intervention. Although in this paper we focus on breast cancer, the developed data analysis strategy is equally applicable to model other cancers and other human diseases where the lack of time-series data to study system dynamics is a *ubiquitous* problem.

## Results and discussion

### Bioinformatics pipeline for cancer progression modeling

A cancer progression path can be mathematically viewed as a complex manifold with multiple branches embedded in a high-dimensional genomic space. Our working hypothesis is as follows: as tumors progress toward advanced stages driven by genetic mutations, each static sample leaves a genetic footprint on the path. If the number of samples is sufficiently large, the footprint information collectively would enable us to recover the progression path and thereby to reveal key genetic events that drive the dynamic evolution of cancer to malignancy. To verify the hypothesis, we developed a novel data analysis strategy to analyze and integrate molecular profile data into a model of cancer progression, which is detailed below.

### Supervised learning approach to identifying cancer progression related genes

Since it is likely that only a small fraction of genes are involved in the malignant process, the first step towards constructing a model is to identify genes associated with cancer progression. This problem has been extensively studied in the community in different contexts [10-12]. One of the most commonly used approaches is correlation analysis [13,14]. A gene with its expression levels highly correlated with survival time is likely to play a role in cancer development. However, correlation analysis can only find genes with a linear dependency with survival time. Moreover, by analyzing one gene at a time, it ignores possible interactions among genes. In molecular classification, a commonly used strategy is first to partition patients into a bad or good prognostic group at a predefined end point (usually 5-year survival or time to metastasis) and then perform feature selection for classification analysis [10-12,15,16]. A major drawback to that approach is that patients with survival times slightly longer or shorter than the end point are put into two different groups. To explore the magnitude information of response variables, we proposed to select relevant genes within a regression framework. The idea has been widely used in the statistics and oncology communities. There are a number of excellent algorithms exemplified by Lasso [17] and its variants, which operate under a linear-model assumption; however, the dependency between gene expression changes and disease progression is complex and is unlikely to be linear [18]. It is thus more appropriate to formulate it as a feature-selection problem for nonlinear regression. However, due to the difficulties of mathematically describing complex data structures, feature selection for nonlinear regression remains a challenging problem.

We developed a new method to address the aforementioned challenge. It is a natural extension of our previous work on feature selection for high-dimensional classification problems [19,20]. The basic idea is to decompose a nonlinear regression problem into a set of linear classification problems and learn feature relevance within a large-margin framework. The development of the algorithm is based on a key observation that a classification problem can be viewed as a degenerated regression problem. To see this, let ${D} = \{(\textbf {x}_{n}, y_{n})\}_{n=1}^{N}\in {\mathcal {R}}^{J}\times {\mathcal {R}}$ be a set of training data, where **x**_*n*_ is the *n*-th sample, *y*_*n*_ is the corresponding survival time, and *J*≫*N*. Without loss of generality, we assume that *y*_*n*_≥*y*_*m*_ if *n*>*m*. If we divide the dataset into two subsets at a predefined end point (e.g., 5 years), we end up with a molecular classification problem. As noted above, a major issue with molecular classification is that it ignores the magnitude information of survival times. Then, a natural idea is to partition the data into two parts at multiple end points $S=\{s_{k} \}_{k=1}^{K}$. A possible choice is *s*_*n*_=(*y*_*n*−1_+*y*_*n*_)/2 for 2≤*n*≤*N*. We seek to select a feature subset so that the overall classification error of unseen test data computed at all end points is minimized. We give below a detailed mathematical description of the proposed method. For the ease of presentation, at the moment, we only consider sample **x**_*n*_ and divide the data into two parts *D*_1_={**x**_*j*_|*y*_*j*_<*s*_*k*_,1≤*j*≤*N*} and *D*_2_={**x**_*j*_|*y*_*j*_>*s*_*k*_,1≤*j*≤*N*} at end point *s*_*k*_. We further assume that *y*_*n*_>*s*_*k*_. We start by defining a margin for **x**_*n*_. Given a distance function, we find two nearest neighbors for **x**_*n*_ from *D*_1_ and *D*_2_, denoted as NN(*D*_1_) and NN(*D*_2_), respectively. The margin of **x**_*n*_ is then defined as *ρ*_*n*_(*s*_*k*_)=*d*(**x**_*n*_,NN(*D*_1_))−*d*(**x**_*n*_,NN(*D*_2_)), where *d*(·) is a distance function. In this study, we used the block distance to measure the similarity between two samples, which was also used in [19,21]. The above margin is called the hypothesis margin, and can be interpreted as a measure of how much **x**_*n*_ can be corrupted by noise before being misclassified by a one-nearest-neighbor classifier [22]. By the large margin theory [23,24], a learning algorithm that minimizes a margin-based error function usually generalizes well on unseen test data. Then, a natural idea is to scale each feature, and thus obtain a weighted feature space, parameterized by a nonnegative vector **w**, so that a margin-based error function in the induced feature space is minimized. The magnitude of each element of **w** reflects the importance of the corresponding feature. The margin of **x**_*n*_, computed with respect to **w**, is given by $\rho _{n}(s_{k}|\textbf {w})= \textbf {w}^{T} (|\textbf {x}_{n}-\text {NN}({D}_{1})|-|\textbf {x}_{n}-\text {NN}({D}_{2})|) \triangleq \textbf {w}^{T} \textbf {z}_{n} (s)$, where |·| is an element-wise absolute operator. Note that *ρ*_*n*_(*s*_*k*_|**w**) is a linear function of **w**, and the margin thus defined requires only information about the neighborhood of **x**_*n*_, while no assumption is made on the underlying data distribution. The above analysis can be repeated for each sample at each end point, leading to the following optimization problem:

(1) $$ \min_{\textbf{w}\ge 0}\sum\limits_{n=1}^{N}\sum\limits_{k=1}^{K}\text{I}(\rho_{n} (s_{k} |\textbf{w})<0)\;,  $$

where I(·) is the indicator function. Since in the inner summation **x**_*n*_ is held out as a test sample, the above formulation can be interpreted as finding a feature subspace where the overall leave-one-out cross-validation error is minimized.

The main problem with the above margin definition is that the nearest neighbors of a given sample are unknown before learning. With tens of thousands of irrelevant features, which is the case in this study, the nearest neighbors defined in the original space can be completely different from those in the induced space. A possible way to address this issue, proposed by [19], is to use a probabilistic model where the nearest neighbor of a given sample is treated as hidden variables. Following the principles of the expectation-maximization (EM) algorithm [25], we estimated the margin by computing the expectation of *ρ*_*n*_(*s*_*k*_|**w**) via averaging out the hidden variables:

(2) $$ {\fontsize{9}{12}\begin{aligned} \bar\rho_{n}(s_{k}|\textbf{w})&=\mathbb{E}[\!\rho_{n}(s_{k}|\textbf{w})]\\ &= \textbf{w}^{T} \left(\sum\limits_{j\in {\mathcal{M}}_{1}}Q(j|n,\textbf{w})|\textbf{x}_{n}-\textbf{x}_{j} |\right.\\ &\left.\quad-\sum\limits_{j \in {\mathcal{M}}_{2}}P(j|n,\textbf{w})|\textbf{x}_{n}-\textbf{x}_{j} |\right)\triangleq\textbf{w}^{T} \textbf{z}_{n}(k)\;, \end{aligned}}  $$

where $\mathbb {E}[\!\cdot ]$ is the expectation operator, ${\mathcal {M}}_{1} = \{j: \textbf {x}_{j}\in {D}_{1}\}$, ${\mathcal {M}}_{2} = \{j: \textbf {x}_{j}\in {D}_{2}\}$, and *Q*(*j*|*n*,**w**) and *P*(*j*|*n*,**w**) are the probabilities of **x**_*j*_ being the nearest neighbors of **x**_*n*_ in *D*_1_ and *D*_2_ with respect to **w**, respectively. The probabilities *Q*(*j*|*n*,**w**) and *P*(*j*|*n*,**w**) can be estimated using the standard kernel method.

After the margins are defined, the problem of learning feature weights can be solved within the large margin framework [23,24]. Note that the indicator function is non-differentiable and non-convex. A commonly used practice to address this issue is to minimize the upper bound of the cost function. We used the hinge loss, which leads to a support vector machine formulation of feature selection for nonlinear regression:

(3) $$ {\fontsize{9}{12}\begin{aligned} \min\limits_{\textbf{w}} \!\sum\limits_{n=1}^{N}\sum\limits_{k=1}^{K} \max\left(\!0,1- \textbf{w}^{T} \textbf{z}_{n}(k)\right)\!, \text{subject to}\, \|\textbf{w}\|_{1}\le \lambda,\textbf{w}\ge \textbf{0}\,, \end{aligned}}  $$

where we imposed an *ℓ*_1_ penalty on **w** to obtain a sparse solution, and *λ* is a regularization parameter controlling the sparseness of a solution, which can be estimated through cross-validation.

Note that **z**_*n*_ implicitly depends on **w** through *Q*(*j*|*n*,**w**) and *P*(*j*|*n*,**w**). We used a fixed-point recursion method to solve **w**. First, we made a guess for **w**, which was used to calculate pairwise distances *d*(**x**_*i*_,**x**_*j*_) and probabilities *Q*(*j*|*n*,**w**) and *P*(*j*|*n*,**w**). Then, the feature weight vector **w** was updated by solving the *ℓ*_1_ regularized optimization problem. The two steps were iterated until convergence. We used our recently developed gradient-descent-based algorithm to solve the above optimization problem efficiently [26]. By using the fixed-point theory [27], it can be proved that the algorithm converges to a unique solution regardless of the initial weights if the kernel width is properly selected. The detailed mathematical derivations are given in the supplementary data, where we also present a simulation study that demonstrates the effectiveness of the new approach.

### Two-pronged approach to cancer progression modeling

**Figure 2:**
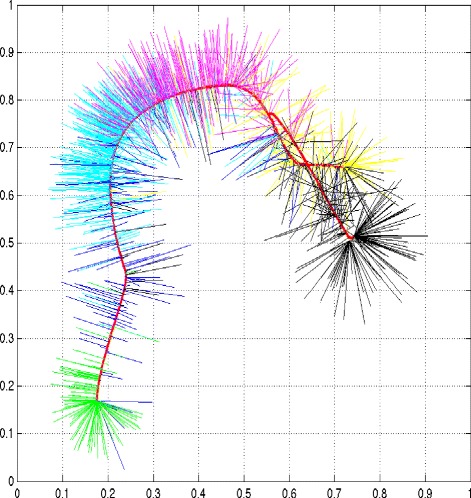
**Visualization analysis of the METABRIC dataset using principal component analysis that gives a general view of sample distribution supported by the selected genes and copy numbers.** The samples were projected onto a principal curve (the red line) constructed using the first three principal components. Each sample was annotated based on its corresponding PAM50 label. Green, normal; blue, normal-like; cyan, luminal A; magenta, luminal B; yellow, HER2+; black, basal. The 144 normal breast tissue samples were used as the baseline. They are first connected with normal-like and luminal A samples, gradually transit to luminal B and form a bifurcation structure leading to basal and HER2+. The 3D plot can be viewed interactively in Adobe Acrobat by setting Adobe Acrobat -> Preference -> 3D & Multimedia.

**Figure 3:**
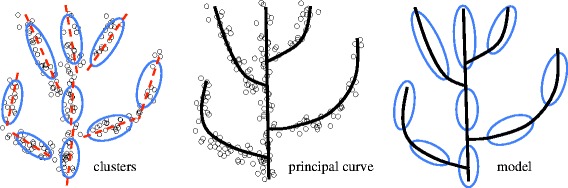
**A toy example illustrating the proposed two-pronged approach.**

After cancer progression related genes are selected, the next step is to build a progression model. Both cancer evolution theory and our data visualization analyses (shown below) suggest that a progression path and clonal structures are two sides of the same coin (see Figure two in [1], Figure 2 and Additional file 1: Figure S13). This motivated us to develop a novel two-pronged method for modeling the cancer progression process. The basic idea is to perform a clustering analysis to detect genetically homogeneous tumor groups, construct a principal curve to summarize the general trend of data, and finally combine the two results using the principal curve as a backbone to build a model of cancer progression. A toy example is given in Figure 3, which illustrates the proposed method.

#### Clustering analysis

Clustering analysis has been intensively studied in the machine learning community. For the purpose of this study, we used spectral clustering. Compared to model-based methods (e.g., *K*-means), spectral clustering does not explicitly impose a model assumption on data distribution, and thus is able to detect clusters of unknown shapes [28]. Moreover, by making use of the spectrum of the similarity matrix of the data, spectral clustering provides an embedded mechanism of dimensionality reduction, making it particularly suitable for this study to cluster high-dimensional data.

Spectral clustering starts with the generation of a similarity matrix measuring the relative similarity of each pair of samples in the dataset. To this end, we first constructed a mutual *K*-nearest-neighbor (KNN) graph based on the profiles of selected genes and copy numbers [28]. On the resulting graph, each vertex represents a sample, and two vertices are connected if the corresponding samples are among the KNNs of each other. Throughout the paper, *K* was set to 30. We tried different values of *K*, and they yielded similar results as determined by the normalized mutual information scores [29]. Based on the constructed KNN graph, a similarity matrix **S** was generated, where the *ij*-th element *S*_*ij*_= exp(−∥**x**_*i*_−**x**_*j*_∥^2^/(*σ*_*i*_*σ*_*j*_)) if **x**_*i*_ and **x**_*j*_ were connected and 0 otherwise. Here, $\sigma _{i} = \|\textbf {x}_{i}- \mathbf {x}_{i}^{(K)}\|$ and $\mathbf {x}_{i}^{(K)}$ is the *K*-th nearest neighbor of **x**_*i*_ [30]. Then, a normalized Laplacian matrix was computed as **L**=**D**^−1/2^**SD**^−1/2^, where **D** is a diagonal matrix with ${D}_{\textit {ii}} = \sum ^{n}_{j=1}S_{\textit {ij}}$, and the eigenvectors of the matrix **L** were computed. The number of clusters was determined using the method proposed in [30]. Briefly, for each possible number of clusters *C*, a rotation matrix that best aligns the columns of the top *C* eigenvectors with the canonical coordinate system was calculated, and the optimal number of clusters was determined as the one that resulted in the highest alignment quality score. Let $\textbf {U} \in \mathcal {R}^{N\times C}$ be a matrix containing the top *C* eigenvectors as columns. We formed a new matrix $\textbf {Y} \in \mathcal {R}^{N \times C} $ from **U** by normalizing the rows to norm one [31]. Finally, the *K*-means analysis was performed to group the samples now represented as the rows of the matrix **Y** into *C* clusters.

To identify robust and stable clusters, the technique of resampling-based consensus clustering [32] was used, where *K*-means clustering was repeated 1,000 times and each time 80% of the samples were drawn randomly without replacement from the entire dataset. The results of the 1,000 runs were then aggregated into a consensus matrix, which gives a visual representation of the frequency of two samples being grouped into the same cluster. To assess the clustering robustness further, the silhouette width of each sample was calculated, which is defined as the difference between its average similarity with samples in the same cluster and highest average similarity with samples in different clusters. A cluster with an average silhouette width >0 is considered stable.

#### Principal curve

We used a principal curve to describe the general trend of the data. Mathematically, a principal curve is a smooth curve going through the center of a data cloud [33,34]. A toy example is presented in Figure 3. This concept was first proposed by [33] as a generalization of the first principal component line and required a curve not to intersect itself, which is too restrictive for our application. Presumably, a cancer progression path is a high-dimensional manifold with multiple branches. Although a dozen algorithms have been developed in the past two decades, there is currently no method that can be used effectively to extract a self-intersected curve embedded in a high-dimensional space (see [35] for an excellent review). For the purpose of this study, we developed a new method that addressed some limitations of existing approaches.

We start by describing a probabilistic model responsible for generating a set of random observations ${D} = \{\textbf {x}_{n}\}_{n=1}^{N} \in {\mathcal {R}}^{M}$. We assume that each sample is generated from an unknown curve in a two-step process. First, a point ***μ***_*s*_ is randomly selected from the curve according to a probability density function *p*(*s*), and then a sample **x** is generated from ***μ***_*s*_ corrupted by Gaussian noise ${\mathcal {N}}(\textbf {x}|{\boldsymbol \mu }_{s}, {\boldsymbol \Sigma }_{s})$, where ***Σ***_*s*_ is a covariance matrix and *s* takes a value from a set *Ω*. We herein do not specify any form for *Ω* to reflect the fact that the curve may contain branching structures. Due to the extremely high data dimensionality (*M*=1,140), it is numerically unstable to estimate full covariance matrices. We thus used a simple heuristic by assuming ***Σ***_*s*_=*σ*^2^**I**, where **I** is an identity matrix. Although we may lose some resolution, the simplification can lead to a numerically stable estimation and worked well for our study. Note that the data generalization mechanism is similar to that assumed in Gaussian mixture modeling [36], with the latter using a set of discrete data points placed at the centers of clusters, instead of a curve, to represent the data. Therefore, principal curve fitting can be viewed as a natural extension of Gaussian mixture modeling.

Let ***θ*** be the parameters {***μ***_*s*_,*p*(*s*)}_*s*∈*Ω*_ and *σ* that we aim to estimate. The log-likelihood function of the data is given by

(4) $$ L(D|\boldsymbol\theta) = \sum\limits_{n=1}^{N} \log\int_{s\in \Omega}{\mathcal{N}}(\textbf{x}_{n}|{\boldsymbol{\mu}}_{s}, {\sigma}^{2}\textbf{I})p(s)ds\;.  $$

The parameters can be estimated by maximizing the log-likelihood function using the EM algorithm [25]. A major issue with the maximum likelihood (ML) estimation is that it is an ill-posed problem and can lead to severe over-fitting [34,36]. Specifically, when *σ* goes to zero, the log-likelihood achieves the maximum value of infinity, resulting in a trivial solution where the obtained curve goes through every data point. One possible remedy for the problem is to add a regularization term to the log-likelihood function to encourage curve smoothness [34]. This strategy, however, works only for simple cases where a curve is not self-intersected. Moreover, it is difficult to determine the regularization parameter used to control the trade-off between data fitting and curve smoothness. It has been shown that cross-validation is not a viable method for controlling the complexity of principal curve estimates [37].

We addressed the issue by exploiting the analogy between principal curve fitting and Gaussian mixture modeling discussed previously. Since the estimation of *σ* causes the overfitting problem, we treated *σ* as a user-defined parameter and only performed the ML estimation on ***μ***_*s*_ and *p*(*s*) for a given parameter. Now the problem becomes how to determine the value of *σ*. It can be shown experimentally that with a decrease of *σ*, the curve complexity, which is measured as the total curve length, increases and the data fitting error decreases monotonically (Additional file 2: Figure S9). In our implementation, a continuous curve was discretized into multiple equally spaced segments, and thus the curve length can be directly translated into the number of segments. The problem now becomes how to estimate the optimal number of segments, which is similar to that of estimating the number of clusters in Gaussian mixture modeling. There is a large body of work on this topic in the clustering literature [36,38,39]. In this study, we used the elbow method [39] for parameter estimation. Additional file 2: Figure S9 shows the curve fitting error measured as the sum of squared distances between data points and their corresponding closest points on a curve versus the curve length. The fitting error decreases monotonically as the curve length increases, but at a certain point the decrease flattens markedly. To perform the estimation automatically, we fitted a regression model consisting of two lines to the two arms of the elbow curve and estimated the data variance as the one that generated a curve with a length equal to that at the intersection of the two lines (Additional file 2: Figures S9, S10 and S11). A detailed mathematical derivation is given in the supplementary data, where we also present a discussion of some implementation issues and a simulation study that demonstrated the effectiveness of the proposed method.

The developed bioinformatics pipeline also contains some standard statistical techniques, including principal component analysis (PCA), survival data analysis, polynomial curving fitting and hypothesis testing. We refer the reader to [40,41] for detailed descriptions. The core algorithms of the proposed bioinformatics pipeline are available upon request.

### Breast cancer progression modeling

#### Identification of breast cancer related genes and data visualization

We demonstrated the utility of the developed bioinformatics pipeline by applying it to a large-scale breast cancer dataset generated by the METABRIC project [7]. The dataset contains the expression levels of 25,160 genes and copy number data of 30,566 genes from 1,989 tumor samples, and has clinical follow-up data ranging from 0 to 25 years^a^. Molecular profiles were obtained from surgically excised primary breast tumors. The majority of estrogen receptor (ER)-positive/lymph node (LN)-negative patients did not receive chemotherapy, whereas ER −/LN+ patients did. Additionally, none of the HER2+ patients received trastuzumab. As such, the treatments were homogeneous with respect to clinically relevant groups. The dataset also contains the gene expression data of 144 normal breast tissue samples. To minimize the confounding factor of censoring in selecting relevant features, we removed 845 samples from the tumor-bearing cohort that had clinical follow-up data of less than 10 years in the feature-selection analysis. Gene expression and copy number data of each sample were then stacked together to form a high-dimensional vector, and robust linear scaling [42] was performed on each feature so that the 2% and 98% quantiles were set to 0 and 1, respectively. As such, all features were comparable and outlier data were removed. We then applied our new feature-selection method (described above) to identify genes associated with cancer progression. Due to the use of an *ℓ*_1_ regulation on feature weights (see Equation 3), the method offers an embedded mechanism to remove irrelevant and redundant features by assigning them zero weights. In this study, features with weights larger than 10^−3^ were selected for the downstream analysis. Using the regularization parameter estimated through tenfold cross-validation, the analysis identified a total of 1,140 features including 989 genes selected from differential mRNA expression data and 151 genes from copy number data.

To obtain a general picture of data distribution supported by the selected features, we first performed a data visualization analysis of the entire 2,133 samples in the METABRIC dataset using PCA and KNN graphic analysis (Figure 2 and Additional file 1: Figure S13, Additional file 3: Movie S1). PCA is a commonly used data dimensionality reduction technique, which projects high-dimensional data onto a three-dimensional space where the data geometric structure is least distorted in a least-square sense [36,40]. For ease of presentation and discussion, we annotated each sample with its corresponding PAM50 label. PAM50 is a 50-gene predictor developed from microarray data that classifies breast cancer samples into intrinsic subtypes including normal-like, luminal A, luminal B, HER2+ and basal [43]. We used the 144 normal breast tissue samples as the baseline. From Figure 2 and Additional file 3: Movie S1, we can see that although the normal samples were not used in feature selection, they are first connected with normal-like and luminal A samples, gradually transit to luminal B samples and form a bifurcation structure leading to either basal or HER2+ samples. Basal and HER2+ tumors are known to be the most aggressive breast cancer phenotypes [44,45]. Note that the three leading principal components account only for 28% of the total information as shown in Movie S1, and consequently some detailed structure is bound to be lost. Therefore, we generated a mutual KNN graph to visualize the data (Additional file 1: Figure S13). On the generated graph, each node represents one sample and two nodes are connected if they are among the KNNs of each other. Although a KNN graph is more sensitive to noise than PCA, one advantage of a KNN graph is that the node connectivity can help to reveal data clustering structures and thus it is commonly used as a preprocess step in spectral analysis [28]. The data visualization result appears to align well with the cancer evolution theory that posits that tumor progression is an evolutionary process akin to Darwinian evolution at the organism level, with clones as the equivalent of genetically distinct quasi-species [1-3]. If the theory is valid, a cancer progression tree would consist of a series of connected, bifurcating clusters, and this is what the PCA and KNN graphs illustrate.

### Construction of a breast cancer progression model

**Figure 4:**
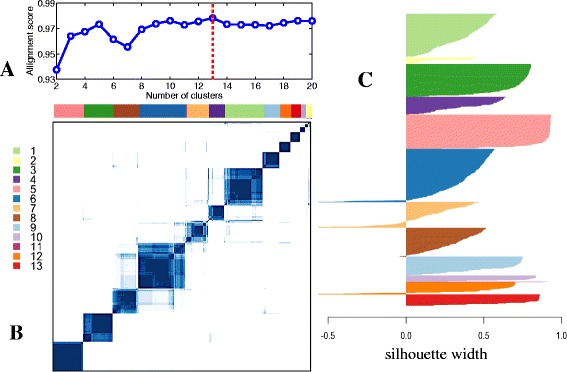
**Spectral clustering analysis performed on the METABRIC data to detect genetically homogeneous tumor groups.** **(A)** The optimal number of clusters was estimated to be 13. **(B)** Resampling-based consensus clustering analysis identified robust and stable clusters. **(C)** Silhouette width analysis was performed to assess the robustness of clustering assignment. The average silhouette width was 0.47.

**Figure 5:**
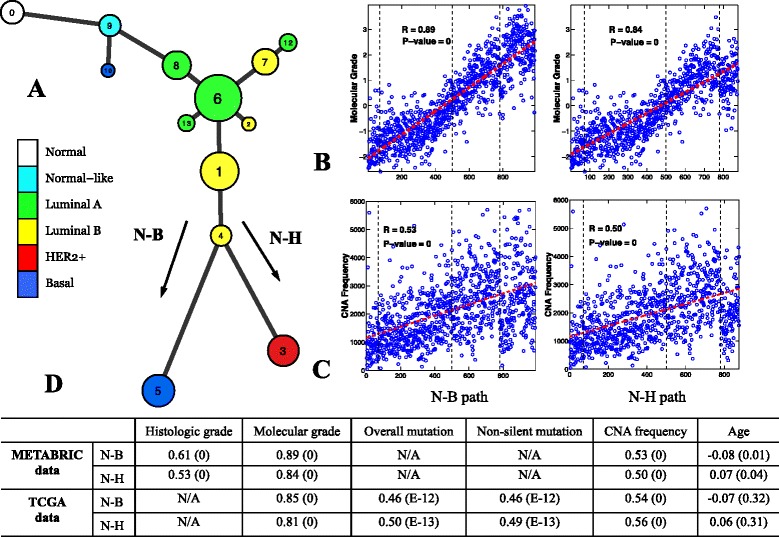
**Model constructed using the METABRIC dataset and its association with clinical and genetic variables.** **(A)** Breast cancer progression tree. Each node represents a cluster and the node size is proportional to the number of samples in the corresponding cluster. Nodes are color-coded based on the PAM50 labels of the majority of the samples in the node. **(B,C)** Molecular grade and CNA frequency were highly correlated with the N-B (first column) and N-H (second column) progression branches. **(D)** Spearman’s rank correlation analysis of histological grade, molecular grade, mutation rate and patient age with the two main progression paths. The numbers in parenthesis are *P* values. CNA, copy number alteration; N-B, normal through luminal to basal phenotype; N-H, normal through luminal to HER2+ phenotype; TCGA, The Cancer Genome Atlas.

The data visualization analysis provided an overview of data distribution, and informed the design of the novel two-pronged method used to model the cancer progression process formally. Application of the spectral clustering method [28] to the METABRIC data revealed 13 distinct clusters (Figure 4A). To promote a robust clustering assignment, a resampling-based consensus clustering analysis [32] was performed. From the generated consensus matrix shown in Figure 4B, we can clearly identify 13 diagonal blocks, which suggests that the clustering assignment is very stable. This result was further confirmed by silhouette width analysis. The clustering analysis classified 1,900 out of 1,989 (96%) samples with a positive silhouette width and yielded an average silhouette width of 0.47 (Figure 4C). Cluster 11 contains only three samples and thus was omitted in downstream analyses. The second step was to extract a principal curve to define mathematically the general trend of the data. To overcome the difficulty of extracting a self-intersected curve embedded in a high-dimensional space, we applied our new principal curve method (described above). The parameter was estimated using the elbow method [39] (Additional file 2: Figure S11). Finally, we combined the clustering and principal curve results using the principal curve as a backbone to build a breast cancer progression trajectory (Figure 5). Each node on the figure represents an identified cluster and the node size is proportional to the number of samples in the corresponding cluster. Two connected nodes indicate a possible inter-relationship, and the length of an edge connecting two nodes is proportional to the distance between the centers of the two nodes. We note that the overall structure of the model is consistent with the results of our data visualization analysis, suggesting that the constructed model faithfully reflects the data distribution.

Our analytical approach revealed a linear, bifurcating structure within the METABRIC dataset. There appear to be two major paths from tumor initiation to malignancy. Intuitively, both paths transition through normal and normal-like tumor phenotypes towards the luminal subtypes. The linear path continues through luminal A and luminal B phenotypes but then bifurcating paths lead from luminal B to either the basal or HER2+ phenotypes (Figure 5A). No evidence for a significant inter-relationship between basal and HER2+ phenotypes was revealed. Beyond the links denoting the major two pathways, we also observe a number of minor side branches. In one case, cluster 9 (normal-like enriched) has a path going directly to cluster 10 (basal enriched) (Figure 5A). Although a small cluster, this suggests that short-cuts to the most malignant phenotypes are possible. Other minor branches emanate from the luminal A node. These represent subdivisions of the major cluster 6 and are in line with the finding that the luminal subtypes are genetically heterogeneous and may have multiple subtypes within them [46,47]. It is tempting to speculate what these minor clusters represent biologically given that the paths appear to be dead ends in an evolutionary context. Perhaps, they represent tumor subtypes that remain dormant or are very slow growing, but this requires further investigation experimentally.

### Survival data analysis

**Figure 6:**
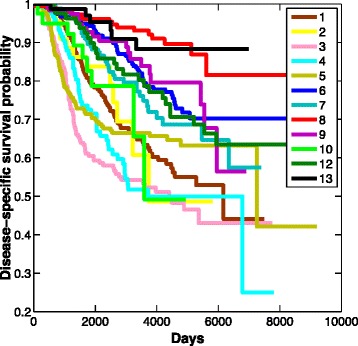
**Overall survival of 12 breast cancer subgroups detected in the METABRIC data.** Cluster 11 contained only three samples and so was omitted.

To examine the relationship between clinical outcome and the groups on the two major paths to malignancy, we used the Kaplan–Meier method [48] to plot overall survival (OS). Figure 6 illustrates a clear trend of worsening survival function associated with progression along the major trajectory through luminal types to basal or HER2+ tumors (cluster 8 through cluster 6, and cluster 1 to either cluster 3 or 5). As would be expected, each cluster, or node, on this linear path generally had a worse OS index than the preceding cluster. Interestingly, cluster 9, located at the start of the linear path between normal samples (no OS data) and the first luminal-type cluster (cluster 8), has a worse OS function than downstream cluster 8. Similar associations with outcome have been reported for this group in other studies [49]. Cluster 9 was classified as normal-like by PAM50 labeling, and there has been conjecture about whether this is an artifact of contamination by high levels of normal tissue in this early stage tumor [50]. The position of the cluster on the progression model may support that notion. A thorough histological investigation of this class of tumors would be needed to resolve this issue. A more plausible explanation is that cluster 9 is connected with cluster 10, which was classed as basal and had a poor OS as shown in Figure 6. This means a subset of tumors in cluster 9 can bypass luminal intermediates and progress to either cluster 10 or 8 directly. Another pattern to note from the Kaplan–Meier plot is the OS data for cluster 4 (Figure 6). This cluster is located at the bifurcation point on the linear model. The OS function for cluster 4 is similar to that for the basal and HER2+ tumors early on, and continues to mimic HER2+ throughout the survival analysis. This implies that pivotal gene activities associated with outcome are acquired at this stage prior to final commitment to a basal or a HER2+ phenotype.

It is worth remembering here that the PAM50 labels were only added to the model after construction to aid in visualization and to help to put the model into context by referral to previous breast cancer classification systems [43]. The analytical approach used in this study identified 13 clusters and revealed associations between them based on statistical significance without any pre-labeling process. Thus, the labels are somewhat misleading and, as shown by the continued subdivision of PAM50 subtypes [46,47,51], do not represent the full complexity of the data structures within tumor tissue molecular profiles. PAM50 labeling works well for the HER2+ and the basal types (clusters 3 and 5 respectively) where the majority of samples in the cluster fit that classification system, but even in these clusters there are admixtures of what would be labeled as basal or HER2+ samples. For the side-branch clusters, the PAM50 admixtures are more pronounced (Additional file 1: Figure S14), nonetheless, the color-coding to some extent aids the visualization of the two major pathways to malignancy in the progression model. If confirmed in independent cohorts, identification of the genetic changes associated with the interconnected clusters revealed in this study may facilitate the refinement of disease classification systems.

As there is currently no established breast cancer progression model for direct comparison, it is important to consider ways to demonstrate the validity of the constructed model. The following sections describe a series of interrogations that provide support for the proposed model and show the utility of such a model for testing and generating hypotheses and providing novel insights. The model revealed two major progression paths; these will be referred to as N-B (normal through luminal to basal phenotype), and N-H (normal through luminal to HER2+ phenotype) in downstream analyses.

### Comparison with conceptual cancer progression models

**Figure 7:**
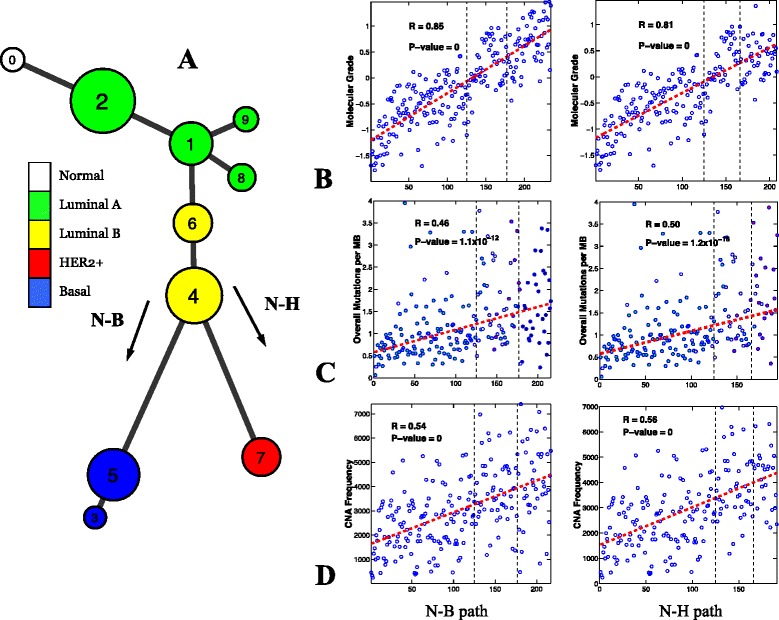
**Model constructed using the TCGA dataset and its association with clinical and genetic variables.** **(A)** Breast cancer progression tree. There were only eight normal-like samples and no distinct cluster was identified. The bifurcation structure and the order of the molecular subtypes are almost identical to those observed in the METABRIC model. **(B-D)** The molecular grade, overall mutation rate and CNA frequency were highly correlated with the N-B (first column) and N-H (second column) progression branches. CNA, copy number alteration; N-B, normal through luminal to basal phenotype; N-H, normal through luminal to HER2+ phenotype; TCGA, The Cancer Genome Atlas.

There have been two major conceptual models proposed regarding the origins of breast cancer subtypes and associated biological mechanisms (see Figure 6 in [47]). One model proposes a distinct-path scenario where each subtype follows a path of initiation and progression independently of the others. The alternative is a linear evolution model, which proposes that tumors gradually evolve from normal cells to malignant states through the accumulation of genetic alterations. While both models embrace the notion of cancer evolution, an important implication from the first model is that the subtypes are considered as different diseases, and the alternative theory proposes that subtypes are different stages of the same disease. Clarifying this issue could have a profound impact as research strategies used in the two scenarios could be very different. The bifurcation structure revealed in our model supports the linear evolution model as a representation of the breast cancer progression process. We should emphasize that our method is a generic approach without making any model assumption on data. If the four major subtypes evolve directly from normal cells, as proposed by the discrete evolution model, in a population study with a large number of samples, we should be able to detect four independent paths connecting normal samples with the four subtypes, but this was not the case (Figures 2, 5 and 7). Our result suggests that basal subtypes are derived from the luminal subtypes, an idea that has been recently suggested through experimentation [52]. The idea that HER2+ phenotypes are derived from luminal B tumors also makes biological sense. Through association of CNA data and putative driver gene expression (data not shown), we found that the copy numbers of the genes involved in the HER2 signaling pathway are significantly amplified in HER2+ samples relative to luminal B samples, suggesting that the HER2+ phenotype develops from luminal B through gene copy number variation, and that this event is distinct from progression to basal phenotypes. This result echoes recent studies that demonstrated that cancer subtypes are not hard-wired, and genotypes and phenotypes can shift over time [1].

### Mapping of tumor grades onto the progression model

We next investigated how grades would be mapped to the two major branches of the constructed progression model. Histological tumor grade is a measure of the extent of abnormal tumor cell morphology relative to normal cells [53]. Since the determination of histological intermediate-grade tumors is somewhat subjective, a method to derive a molecular grade index has been developed [54], and the information required to derive this index was available in the METABRIC dataset. We found that both the molecular and histological grades were highly correlated with both of the major progression paths. Figure 5B plots molecular grade against the samples on the model’s N-B progression path and reveals that there is a clear increase in grade along the path to the most malignant basal phenotype samples. Likewise, for the N-H path (Figure 5B) a clear trend in molecular grade towards the malignant HER2+ samples was observed. Statistical significance was determined by Spearman’s test. Briefly, each sample was projected onto the specific progression path (the projection of a sample is defined as a point on the path that is the closest to the sample; see Additional file 2: Figures S8 and S10), and then a Spearman correlation analysis of histologic/molecular grade was performed against the N-B/N-H paths. Strong correlation was observed for both molecular grade (N-B: *r*=0.89, *P*=0; N-H: *r*=0.84, *P*=0) and histological grade (N-B: *r*=0.61, *P*=0; N-H: *r*=0.53, *P*=0; see Figure 5D).

The data support the validity of the progression model in that statistically significant correlations were identified, but also because it aligns with established grade associations. Nearly all basal and HER2+ tumor phenotypes are aggressive grade-3 cancers and the majority of luminal A tumors are low grade-1 cancers [55]. In itself, this is difficult to explain within a discrete disease evolution model as it implies that basal and HER2+ tumors are high-grade cancers from the outset. The correlation aligns well with a linear evolution model. Interestingly, the mapping of the grades onto the model also supports a malignancy-associated transition from luminal A to luminal B phenotypes. Luminal B tumors are routinely graded higher histologically than luminal A tumors, and grade-3 rates (and outcomes) approach those seen in the more aggressive HER2+ and basal phenotypes [47,55]. It has been proposed that this phenomenon reflects a yet more complex inventory of subtypes within the luminal B phenotype [47], but it could also be explained by a progressive transition from luminal A to luminal B and then on to the aggressive HER2+ or basal tumor phenotypes.

### Model validation using an independent breast cancer dataset

To investigate whether a similar model would be derived from an independent dataset, we performed a computational analysis on data obtained from The Cancer Genome Atlas (TCGA) breast cancer project [8]. As such, we have used data from the two most comprehensive breast cancer projects conducted to date. In the TCGA project, a total of 507 tumor tissues and 17 normal tissues were subjected to gene expression and copy number profiling. It is often difficult to perform direct comparisons between large projects because the data sources are not entirely compatible. In this case, the TCGA study employed different microarray platforms, and the clinical follow-up was markedly shorter (median overall follow-up was 17 months vs 98 months for the METABRIC data, and there were only 90 overall survival events), so feature selection on this dataset would be underpowered. Instead, we mapped the genes selected from the METABRIC data analysis back to the TCGA data to perform the clustering and principal curve analyses and model construction. A total of 775 of the 989 genes selected from the METABRIC dataset were also found in the TCGA data. On application of the same two-pronged analytical protocol, nine stable clusters were identified (Additional file 1: Figures S15 and S16). The difference in the numbers of clusters found in the two studies can be attributed to various factors including the different sample sizes, the different microarray platforms used, and the partially overlapped gene sets. Despite those differences, the major structures of the progression model constructed using the TCGA data (i.e., the bifurcation structure and the order that the clusters are connected) were almost identical to those constructed using the METABRIC data (Figure 7). As with the METABRIC data model, luminal A clusters represented the largest group in the cohort, there were four luminal A type and two luminal B type clusters in series, and there were some dead-end side branches emanating from the luminal phenotypes. Because only 8 out of 507 tumor samples were classified as normal-like, no distinct cluster was identified on the TCGA progression tree. Survival curves for the clusters in the TCGA model could not be constructed due to the lack of follow-up data. Consistent with the results obtained from the METABRIC progression model, the progression model constructed using the TCGA data was also significantly correlated with the molecular grade index (Spearman’s test, N-B: *r*=0.85, *P*=0; N-H: *r*=0.81, *P*=0). Of note, the magnitudes of the correlation obtained for both METABRIC and TCGA models were comparable (see Figure 5D). No histological grade data was available for the TCGA data.

### Mapping genetic alterations onto the progression models

A bonus of the TCGA project was the availability of the overall and non-silent mutation rates for each sample. This information, together with the frequency of CNAs, was used to test model validity. Here, the CNA frequency of a sample is defined as the sum of the magnitude of CNAs including amplification and deletion of all genes in the sample. It is widely accepted that cancer development is due to the accumulation of genetic alterations in somatic cells [1-4]. Among them, somatic point mutations and CNAs play a central role in tumorigenesis [56,57]. If our constructed breast cancer progression models are valid, we would expect somatic mutation rates and CNA frequencies to be positively correlated with the modeled progression trajectory. To investigate this, we mapped the overall mutation rate and CNA frequency of each sample back to the two major progression branches revealed by the TCGA model and found that this is *indeed* the case for both somatic mutation rate (N-B: *r*=0.46, *P*=1.1×10^−12^; N-H: *r*=0.50, *P*=1.2×10^−13^; see Figure 7C) and CNA frequency (N-B: *r*=0.54, *P*=0; N-H: *r*=0.56, *P*=0; see Figure 7D). A similar result was observed for non-silent mutation rates (Figure 5D). We also found that patient age had no significant correlation with the progression model (Spearman’s test, N-B: *r*=−0.07, *P*=0.32; N-H: *r*=0.06, *P*=0.31; see Figure 5D)^b^. This finding means that the significant correlation between genetic alteration rates and the two major progression branches are statistically independent of patient ages. This is in line with recent findings that showed that although tumors originating from many self-renewing tissues have increasing mutation rates with age, no such correlation has been found in breast or ovarian cancer [58]. Consistent with the results obtained from the TCGA model, the progression model constructed using the METABRIC data was also significantly correlated with the CNA frequency (Spearman’s test, N-B: *r*=0.53, *P*=0; N-H: *r*=0.50, *P*=0; see Figure 5C,D). No somatic mutation data were available for the METABRIC data.

Genome instability is generally referred to as an enabling characteristic of cancer progression [5]. The significant correlation of both somatic mutation rate and CNA frequency in the progression models built from two independent datasets provides strong evidence supporting the validity of the proposed model.

### Mapping of key genes onto breast cancer progression paths

**Figure 8:**
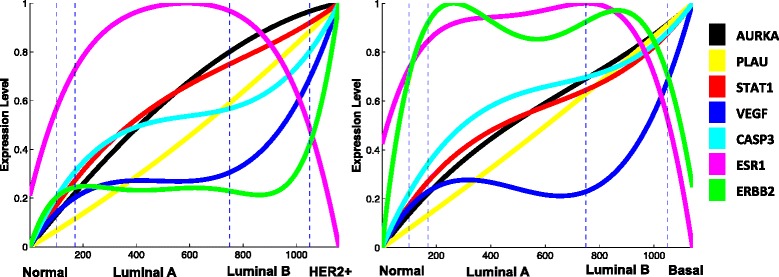
**Seven key genes (AURKA, PLAU, STAT1, VEGF, CASP3, ESR1, and ERBB2) representing proliferation, tumor invasion/metastasis, immune response, angiogenesis, apoptosis phenotypes, and ER and HER2 signaling, respectively, were mapped onto the two major progression branches of the METABRIC model.** For ease of presentation in one plot, the expression level of each gene was normalized into the interval of [0,1]. The small interval between normal and luminal A represents normal-like.

Next, we investigated the change in gene expression of some key genes during breast cancer progression by mapping them onto the model derived from the more comprehensive METABRIC dataset. Our initial interrogation mapped the expression of seven key genes (AURKA, PLAU, STAT1, VEGF, CASP3, ESR1 and ERBB2), which represent described hallmarks of cancer, namely proliferation, tumor invasion and metastasis, immune response, angiogenesis, apoptosis, and estrogen (ER) and HER2 signaling, respectively [59]. The resulting plots revealed the change of expression of these genes along both linear paths, normal to HER2+ (N-H, Additional file 1: Figure S17) and normal to basal (N-B, Additional file 1: Figure S18). By normalizing the expression levels, we were able to visualize the changes on a single overlay plot (Figure 8). The curves were generated using the polynomial curve fitting method and the degree parameter was estimated through tenfold cross-validation [40]. Once again, we label the axis with PAM50 subtype labels to indicate an approximation of tumor subtypes for ease of discussion.

As would be expected, the ERBB2 gene is only highly expressed in the HER2+ cluster on the N-H path (Figure 8, left), suggesting ERBB2 amplification is a late onset event in tumor progression. As also expected, the estrogen receptor ESR1 is low in normal phenotypes, increased in luminal phenotypes that are primarily classified as ER+, and lost in the most malignant basal and HER2+ phenotypes. On the N-B path plot (Figure 8, right), we see that basal phenotypes are ER −/HER2 −. Most triple-negative tumors (ER −/HER2 −/PR −), which carry a particularly poor prognosis, have a basal phenotype [60]. VEGF expression increases from the normal to luminal phenotypes, but then plateaus across luminal phenotypes until the transition to malignant HER2+ or basal phases. This may suggest that angiogenesis is a limiting factor for progression from luminal phenotypes, perhaps indicating a potential point of efficacy for anti-angiogenic therapy. As an indicator of tumor invasion, higher PLAU expression is expected at the malignant disease stages, and this is observed for both progression paths, but the correlation is less pronounced than for other indicators. The plots reveal that increasing AURKA expression is very strongly correlated with progression to malignancy. Intuitively, we might expect that proliferation will increase in an overall sense as cancer develops, but even malignant tumors are relatively slow-growing from a cellular mass point of view, and so tumors have to overcome opposing events to achieve a net growth. This is supported by the fact that the expression of CASP3, an indicator of apoptosis [61], also increases in parallel with AURKA as tumors progress. AURKA has been reported to be associated with outcome as the key gene in a proliferation module [59] and as part of a three-gene molecular signature for subtype classification of tumors [62]. Those studies provided support for AURKA gene expression as a classifier or a biomarker for prognosis using associations with particular subtypes, but the positive correlation across N-H and N-B paths revealed by plotting the progression model provides further support for AURKA monitoring as a powerful biomarker of tumor status and prognosis. Likewise, the strong correlation of expression of STAT1 suggests that this factor could also act as a marker of progression status and outcome. The transcription factor STAT1 has been thought of as an onco-suppressive factor, acting through the stimulation of anti-proliferative and pro-apoptotic genes in tumor cells, but recent evidence supports a multivalent role for this factor in advancing cancer. STAT1 expression is up-regulated in a number of late-stage cancers, including breast cancer [63-65], and high STAT1 levels have been correlated with poor survival, presence of metastases and with chemo- and radiotherapy resistance in breast cancer patients [66,67]. Potential functions of STAT1 in aggressive cancers include the maintenance of pro-survival genes [68] and the regulation of the local immune response [65]. There are conflicting reports of STAT1 expression levels in breast cancer subtypes [59], but in the large METABRIC dataset, STAT1 expression was significantly increased in the malignant phenotypes.

We also explored the potential association of reported cancer driver genes with breast cancer progression using the same model interrogation approach. The 125 driver genes selected have been defined through large-scale mutational analyses [69], but changes in their expression level may also enhance or inhibit some aspect of tumor progression. A total of 31 genes were found to have significant changes in expression across either path of the progression model (Additional file 1: Figures S19 and S20 and Additional file 4: Table S1). From the individual plots, we see that a few genes have a distinctive association with progression. Of note are the opposing curves of EGFR and NOTCH1 versus GATA3 and RET. The former pair have U-shaped curves depicting higher expression in normal and malignant phenotypes, whereas the latter pair have pronounced bell-shaped curves that mirror the EGFR/NOTCH1 expression profile. These genes may have a co-ordinate expression relationship, being regulated by each other, or perhaps through the estrogen receptor, which has a similar profile to GATA3/RET across the progression model, and such networks are beginning to be elucidated [70]. Other notable plots include EZH2, which is expressed at progressively higher levels on both paths to malignancy, and SMAD4 and KIT, which similarly decrease during transition from normal to malignant states. The only driver gene to have a distinct spike in expression in the malignant phenotypes was CDKN2A. This gene encodes the p16INK4a tumor suppressor protein, which regulates the cell cycle, and so the marked increase in expression in both basal and HER2+ phenotypes is intriguing. Interestingly, recent studies have shown that in breast cancer, high expression is indicative of a more undifferentiated phenotype [71], and elevated CDKN2A has been proposed as a marker of poor prognosis [72]. Our progression model supports these recent reports. While the driver gene analyses do not address the mutational status of the specific genes, the level of their expression is of interest because it will impact the potential effect that the driver mutations may have on tumor phenotype.

### Discussion

Overall, our findings support the idea that cancers acquire the properties required for progression towards malignancy from an accumulation of genetic aberrations, a phenomenon that is consistent with clonal evolution theory. This process occurs over extended time periods, measured in years, and so accurate mapping is logistically very challenging. From cancer patients, we typically only have data from a single time point through sampling at the time of surgery, and this restriction is unlikely to be overcome for the majority of solid tumors. While it may subsequently be possible to reconstruct the development of malignancy through a longitudinal study of a few select patients, to overcome issues of heterogeneity, a large number of tissue samples is possibly needed first to construct a theoretical model of progression. This can only be achieved through the analysis of available static samples. A computational approach that can overcome the sampling limitation and thereby enable the leveraging of accumulating data and the vast tissue archive represents a major advance with respect to the application of bioinformatics methodology to the study of progressive human diseases.

There are many potential applications for the proposed approach. One of them is to identify genetic alterations that drive tumor progression, which is one of the central goals of some recent large-scale cancer studies [7,8,73-76]. The primary analytical strategy used today is prevalence-based approaches that search through a large number of tumor samples for genetic changes that occur with a higher frequency than would be expected by chance alone [73,74]. While the associations between genetic events and disease can be revealed by existing methods in an overall sense and can even be associated with a putative tumor subtype, the placement of molecular events onto a cancer progression map through a progression-model-based approach provides a way to put these molecular events into the context of a dynamic disease process and actually enables us to determine what changes are pivotal to transition from one phenotype to another. If confirmed, such a model could provide a foundation for the visualization of key progressive molecular events and facilitate the identification of pivotal driver genes and pathways, the most relevant and robust biomarker signatures, and potential points of susceptibility for therapeutic intervention. Moreover, interrogation of the model will allow researchers to test novel hypotheses *in silico* and thus help to prioritize resources for more focused and detailed investigations experimentally.

The presented study has demonstrated the possibility of using static sample data to study disease dynamics, which lays a foundation for future studies to incorporate all currently available molecular data (e.g., mRNA, microRNA, copy number, methylation and protein expression data) to construct high-resolution cancer progression trees. Our ongoing work will thus focus on addressing the challenges associated with processing massive, high-dimensional data of different types to build models of increasing resolution. This in turn will further facilitate the elucidation of clinically and biologically pertinent issues. The described computational methods can also be further improved accordingly. For example, in the proposed EM-based principal curve-fitting algorithm, we assumed that the data was corrupted by noise with an equal variance throughout the genomic space. This heuristic approach worked well, but to build higher resolution models, we are working to develop an algorithm using data-driven variance estimation. In this study, we used a two-pronged approach consisting of spectral clustering and principal curve to model a cancer progression path. Incorporation of other data dimensionality techniques (e.g., self-organizing map [77] and Isomap [78]) may also aid the production of a low-dimensional representation of the input space of cancer samples. We expect that the results would be similar, but an intensive computation study is needed to assess the utility of other techniques for cancer progression modeling.

## Conclusions

We describe the derivation of a novel computational approach for mapping the development of cancer towards malignancy. Through application to two independent, large-scale breast cancer datasets, we have shown that the proposed method can reconstruct tumor progression through the analysis of static samples and thereby identify genetic events associated with pivotal shifts in phenotype. This new set of tools will enable the construction of high-resolution progression trees for cancers and other diseases for which longitudinal data sampling is ethically or logistically not possible. Refinement of progression trees will facilitate the identification of molecular drivers of disease progression and the derivation of robust biomarker signatures for patient evaluation and management.

## Endnotes

^a^ The data is available in the European Genome-Phenome Archive with the accession number [EGAS00000000083].

^b^ Based on a rule of thumb used in the statistical community, two variables with a correlation larger than −0.2 and smaller than 0.2 are considered as having no or negligible relationship.

## Additional files

Additional file 1
**Supplementary figures.**

Additional file 2
**Supplementary data.** Detailed mathematical derivations of the proposed methods and simulation studies. It also contains Figures S1 to S11.

Additional file 3
**Movie S1.** An overview of the data distribution.

Additional file 4
**Table S1.** Shown are 31 putative cancer driver genes.

## Abbreviations

EM: expectation maximization
ER: estrogen receptor
KNN: *K* nearest neighbor
LN: lymph node
miRNA, microRNA; ML: maximum likelihood
N-B: normal through luminal to basal phenotype
N-H: normal through luminal to HER2+ phenotype
OS: overall survival
PCA: principal component analysis
TCGA: The Cancer Genome Atlas
