# Supplementary material for: Cancer progression modeling using static sample data
Source: Genome Biol. 2014 Aug 26;15(8):440. doi: 10.1186/s13059-014-0440-0 (PMC4196119; doi:10.1186/s13059-014-0440-0)
Supplement: Additional file 1 — Supplementary figures. [file 13059_2014_440_MOESM1_ESM.pptx]

## Slide 1
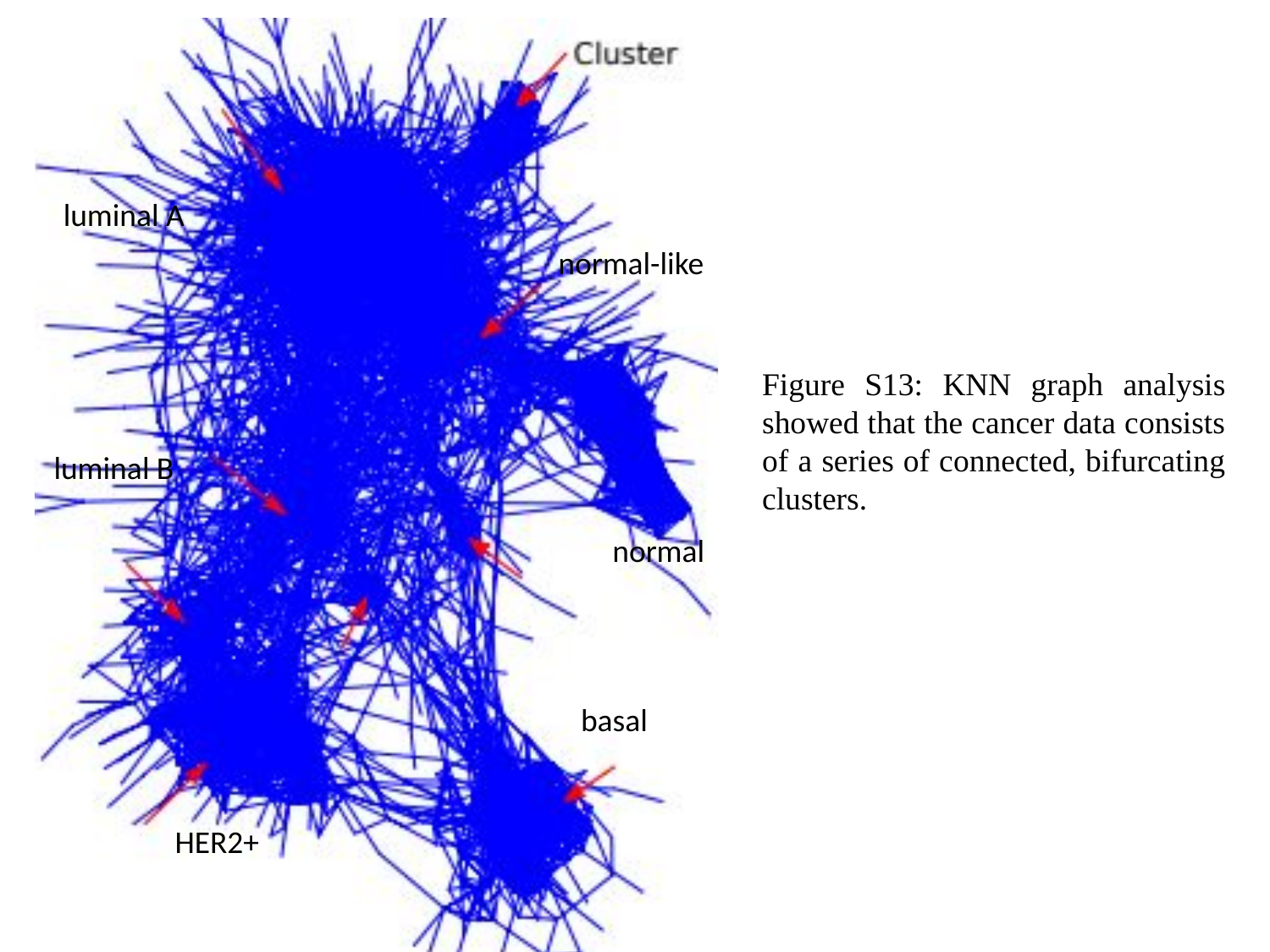

luminal A
normal-like
Figure S13: KNN graph analysis showed that the cancer data consists of a series of connected, bifurcating clusters.
luminal B
normal
basal
HER2+

## Slide 2
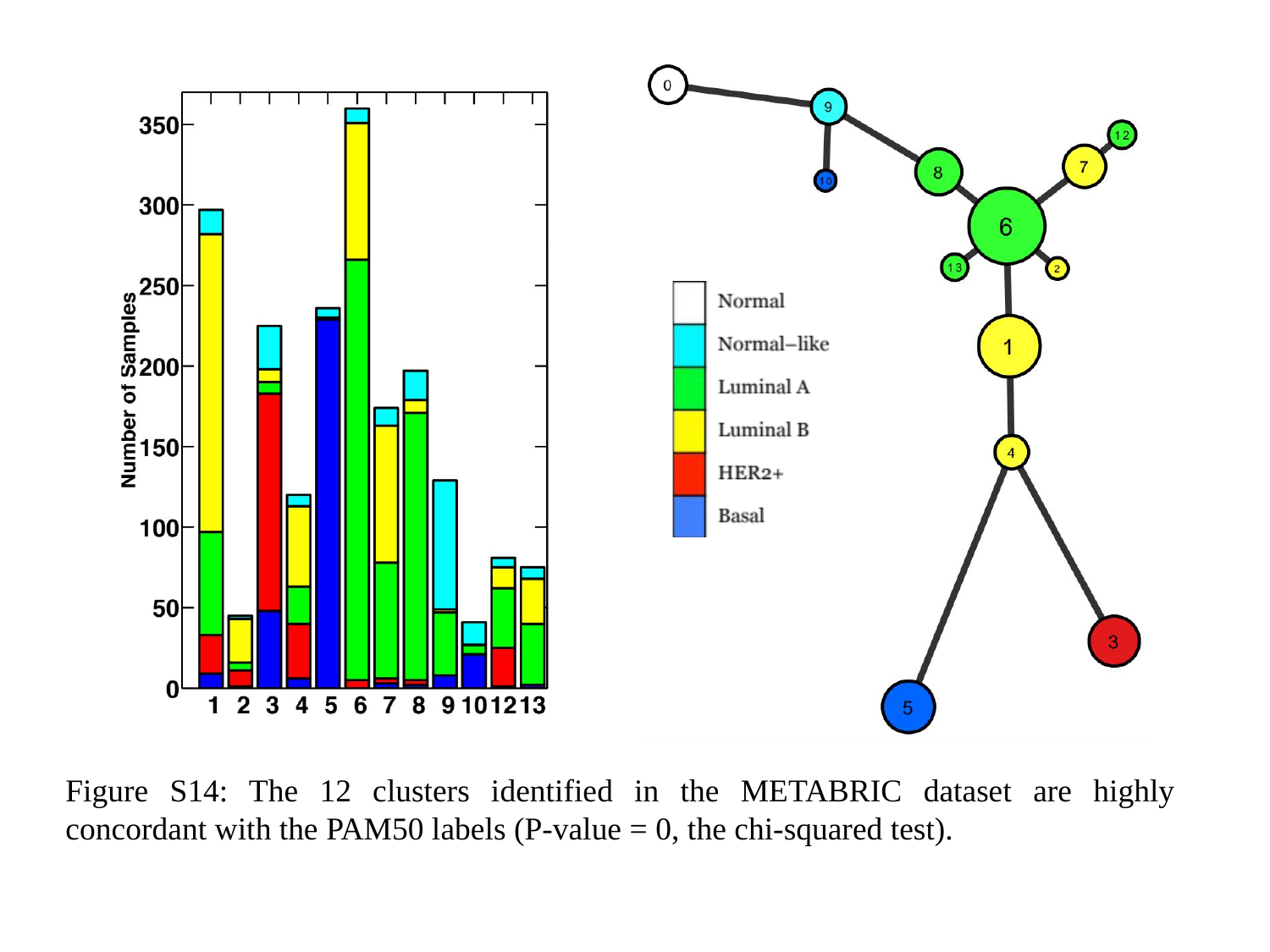

Figure S14: The 12 clusters identified in the METABRIC dataset are highly concordant with the PAM50 labels (P-value = 0, the chi-squared test).

## Slide 3
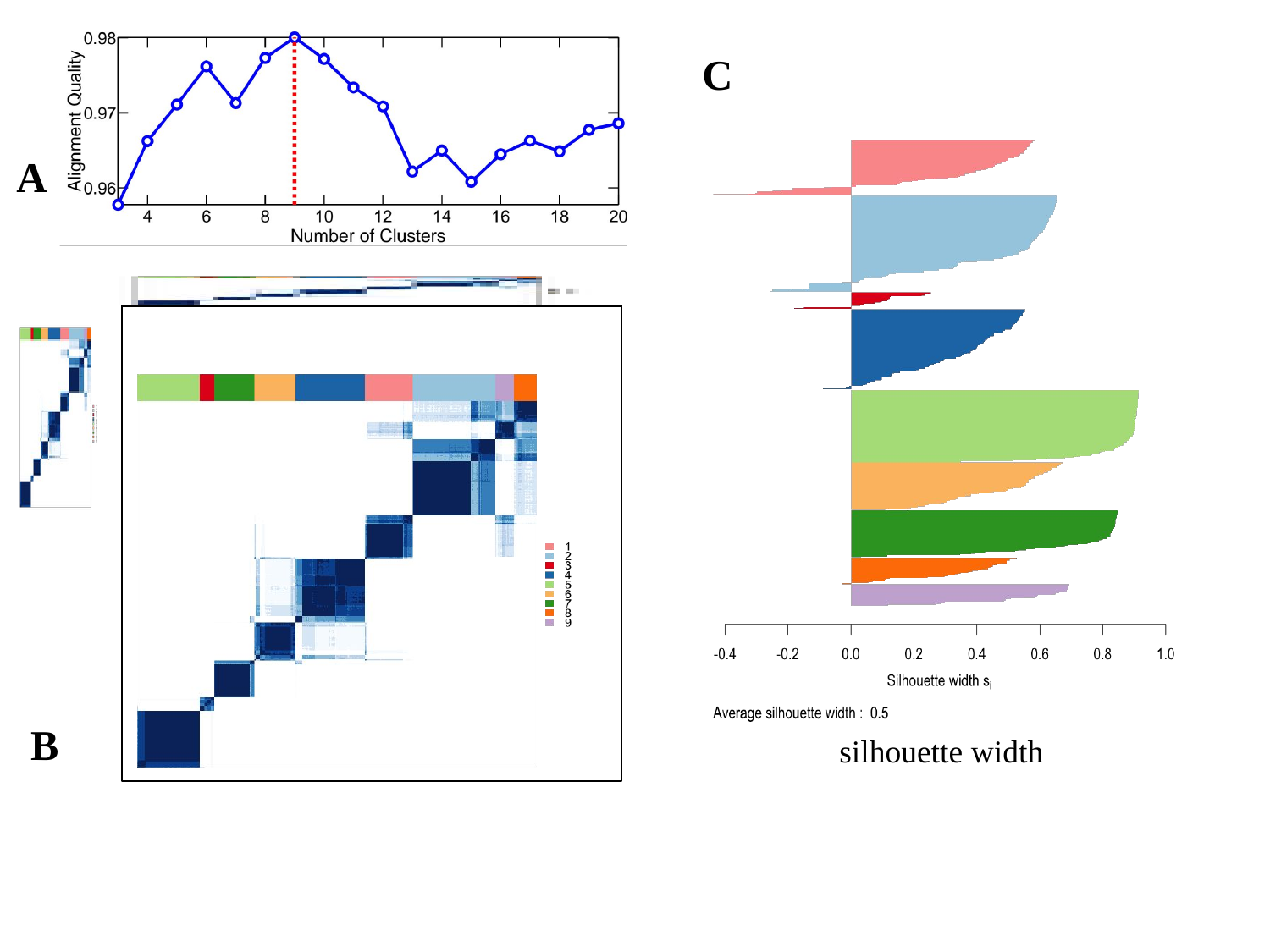

C
A
B
silhouette width

## Slide 4
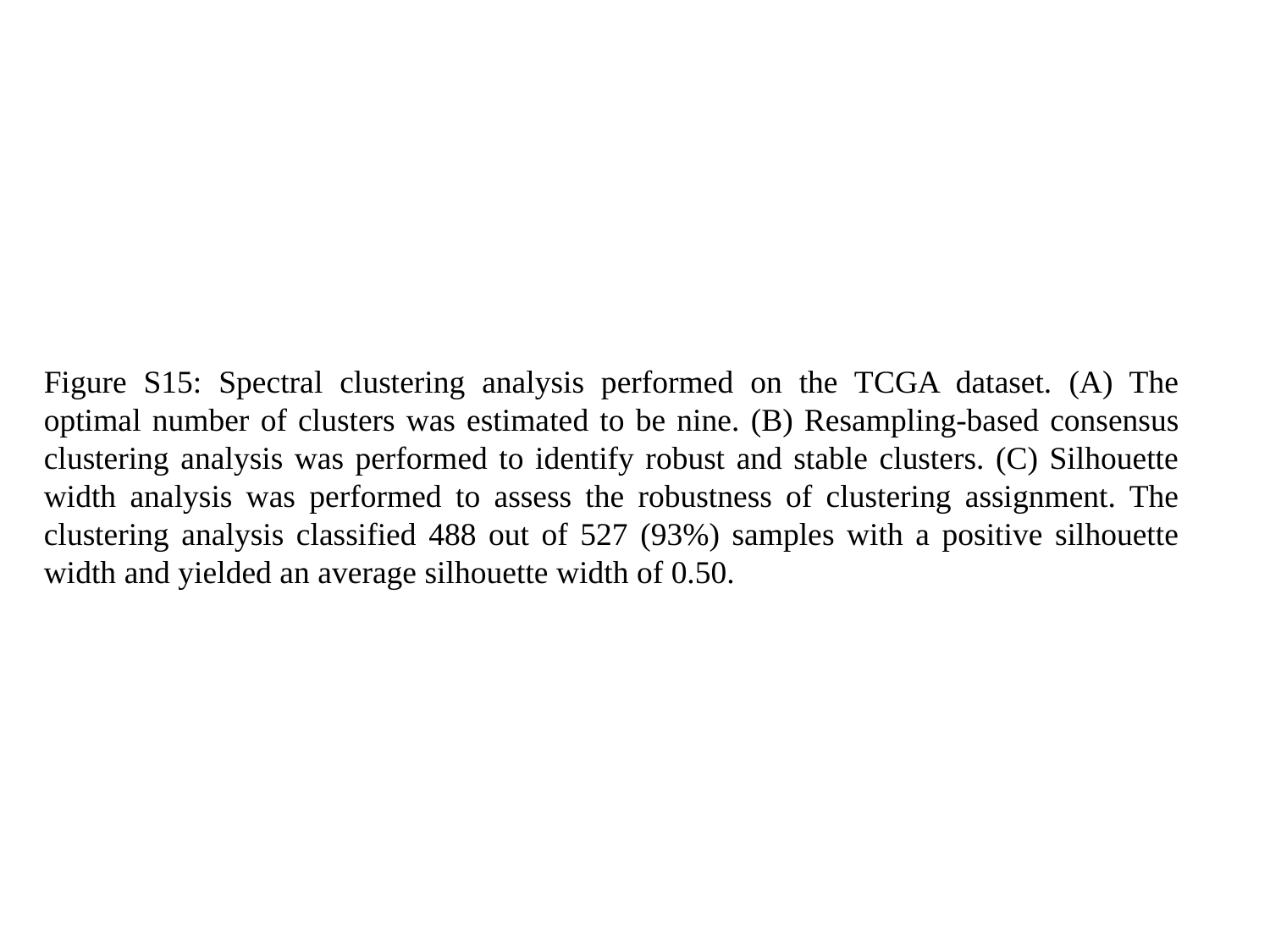

Figure S15: Spectral clustering analysis performed on the TCGA dataset. (A) The optimal number of clusters was estimated to be nine. (B) Resampling-based consensus clustering analysis was performed to identify robust and stable clusters. (C) Silhouette width analysis was performed to assess the robustness of clustering assignment. The clustering analysis classified 488 out of 527 (93%) samples with a positive silhouette width and yielded an average silhouette width of 0.50.

## Slide 5
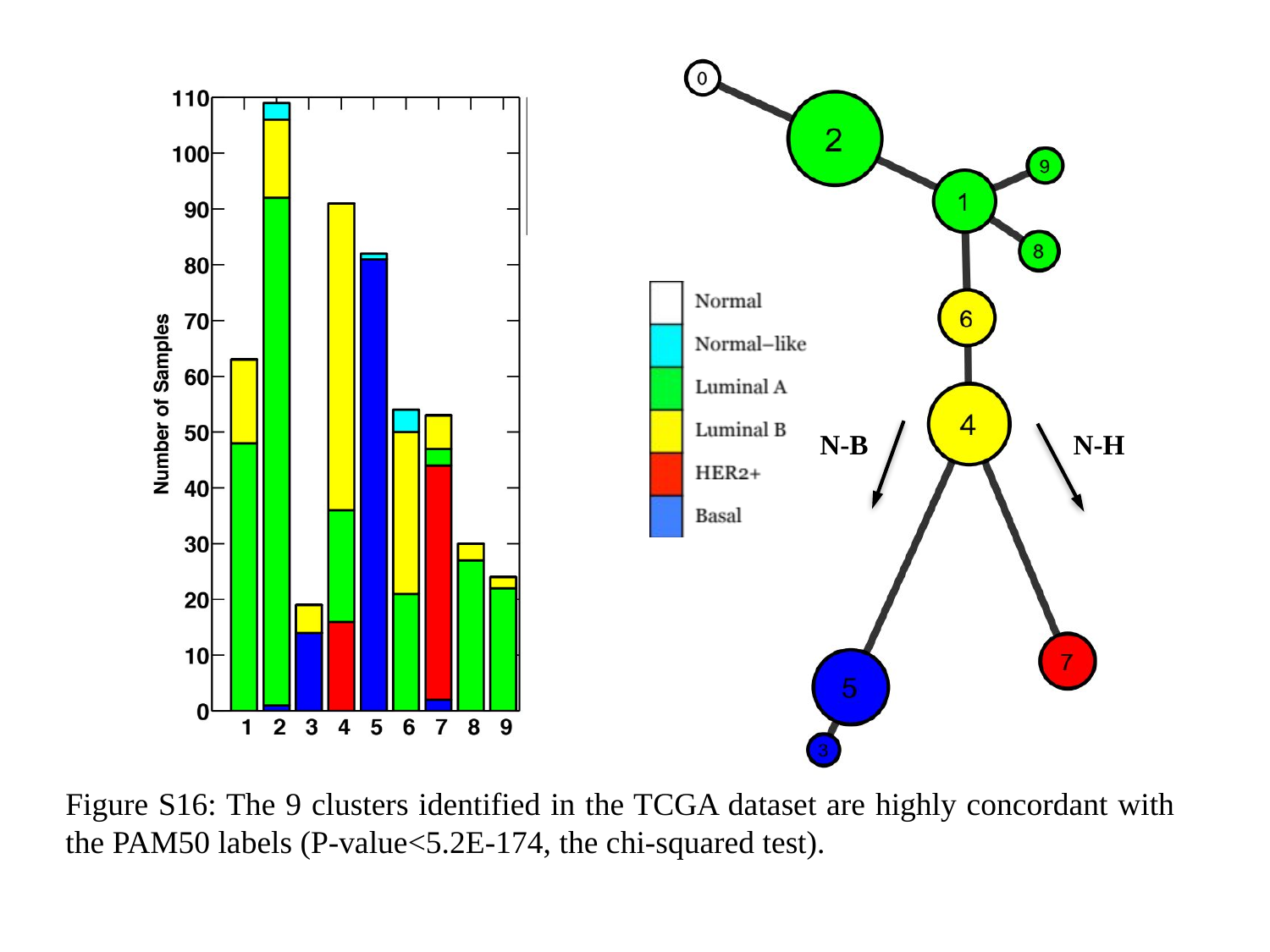

N-B
N-H
Figure S16: The 9 clusters identified in the TCGA dataset are highly concordant with the PAM50 labels (P-value<5.2E-174, the chi-squared test).

## Slide 6
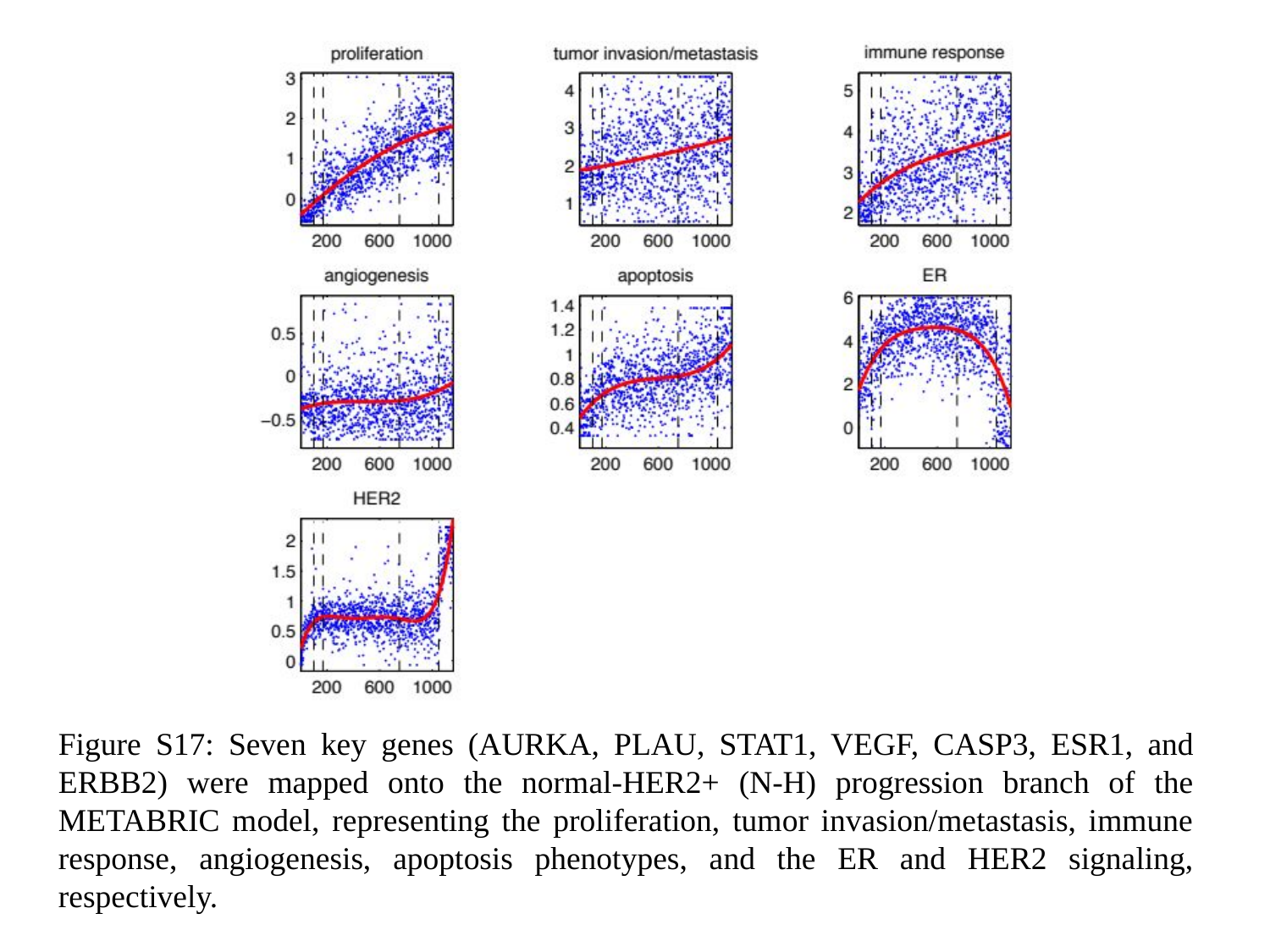

Figure S17: Seven key genes (AURKA, PLAU, STAT1, VEGF, CASP3, ESR1, and ERBB2) were mapped onto the normal-HER2+ (N-H) progression branch of the METABRIC model, representing the proliferation, tumor invasion/metastasis, immune response, angiogenesis, apoptosis phenotypes, and the ER and HER2 signaling, respectively.

## Slide 7
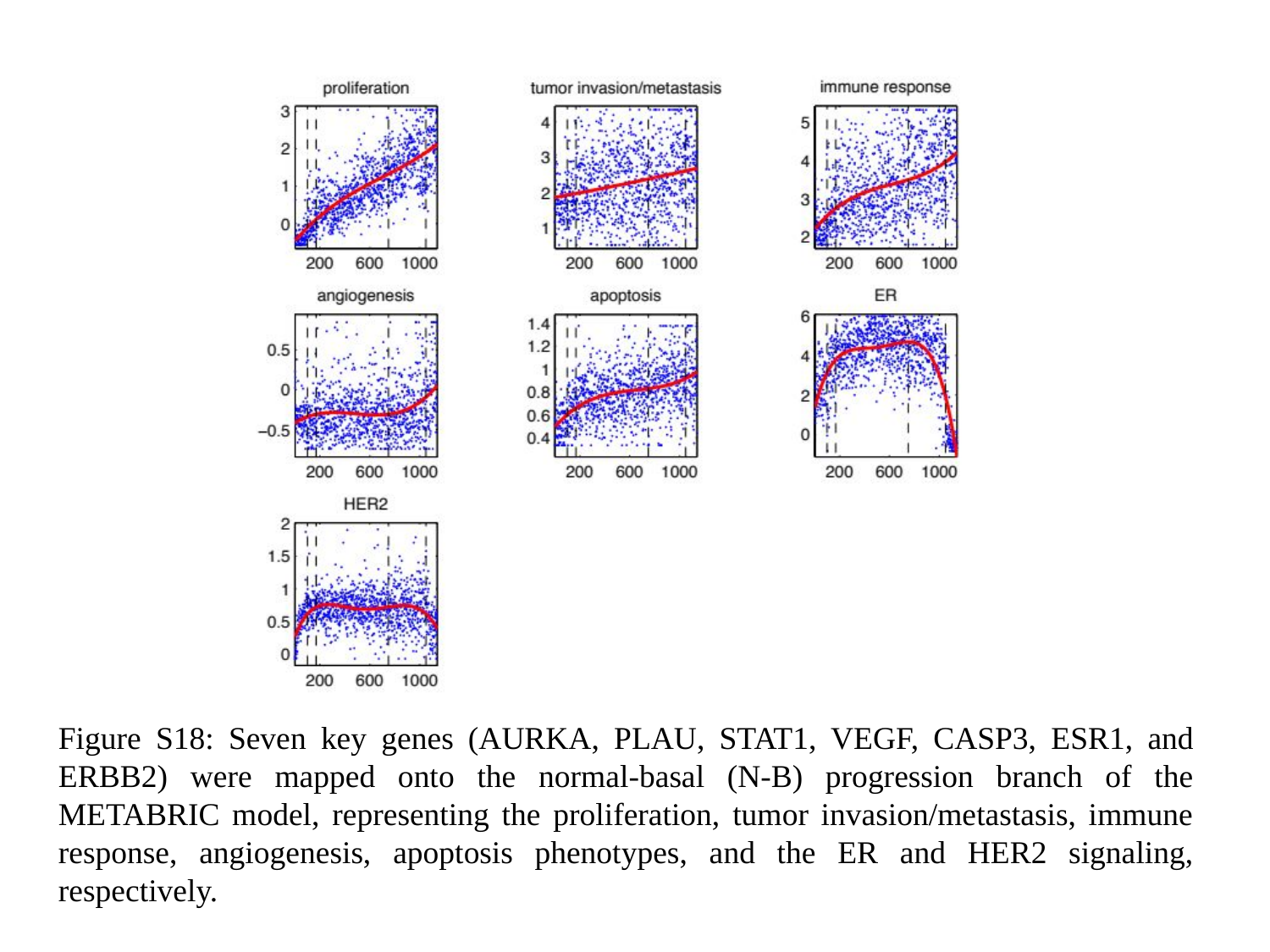

Figure S18: Seven key genes (AURKA, PLAU, STAT1, VEGF, CASP3, ESR1, and ERBB2) were mapped onto the normal-basal (N-B) progression branch of the METABRIC model, representing the proliferation, tumor invasion/metastasis, immune response, angiogenesis, apoptosis phenotypes, and the ER and HER2 signaling, respectively.

## Slide 8
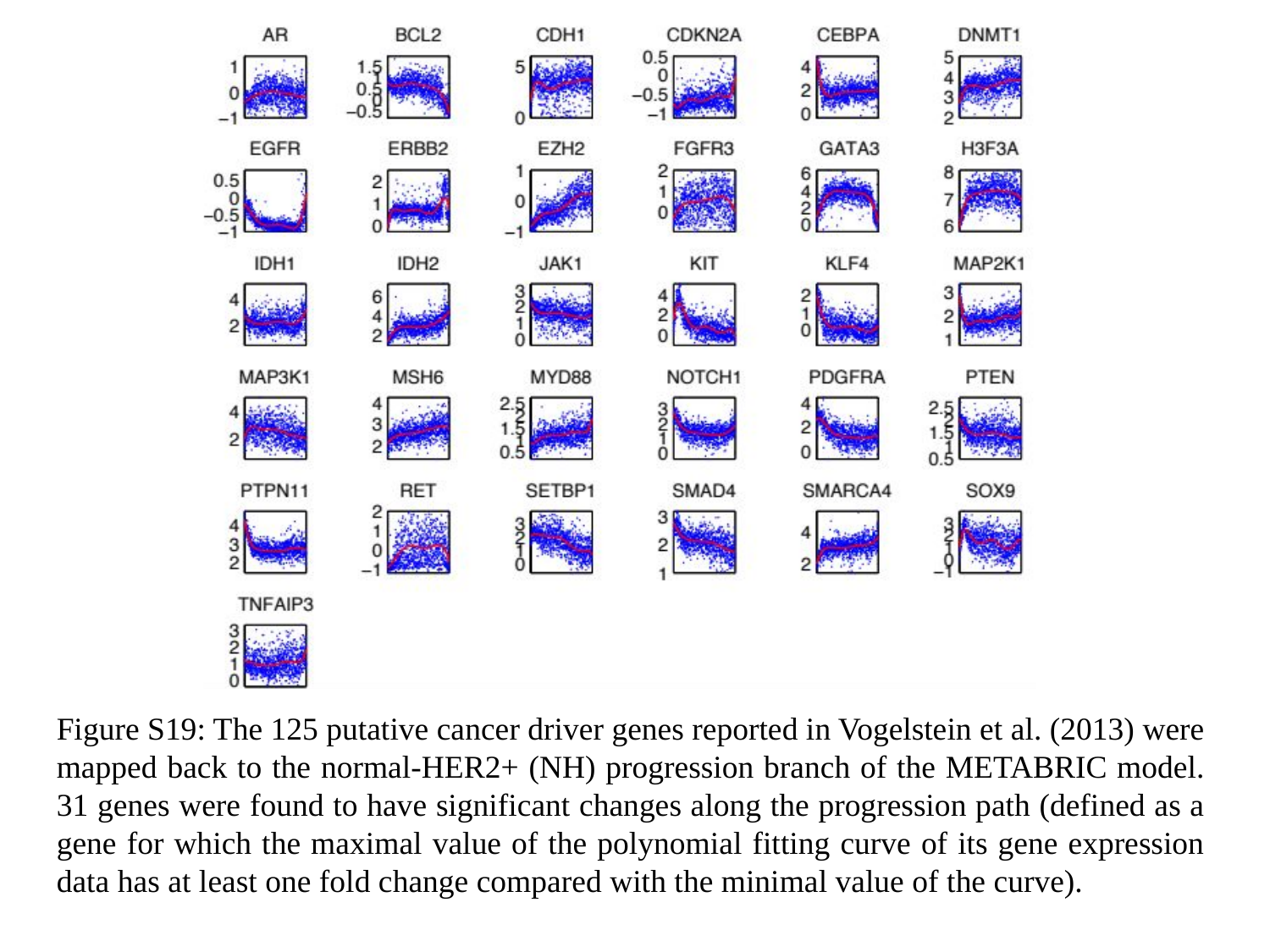

Figure S19: The 125 putative cancer driver genes reported in Vogelstein et al. (2013) were mapped back to the normal-HER2+ (NH) progression branch of the METABRIC model. 31 genes were found to have significant changes along the progression path (defined as a gene for which the maximal value of the polynomial fitting curve of its gene expression data has at least one fold change compared with the minimal value of the curve).

## Slide 9
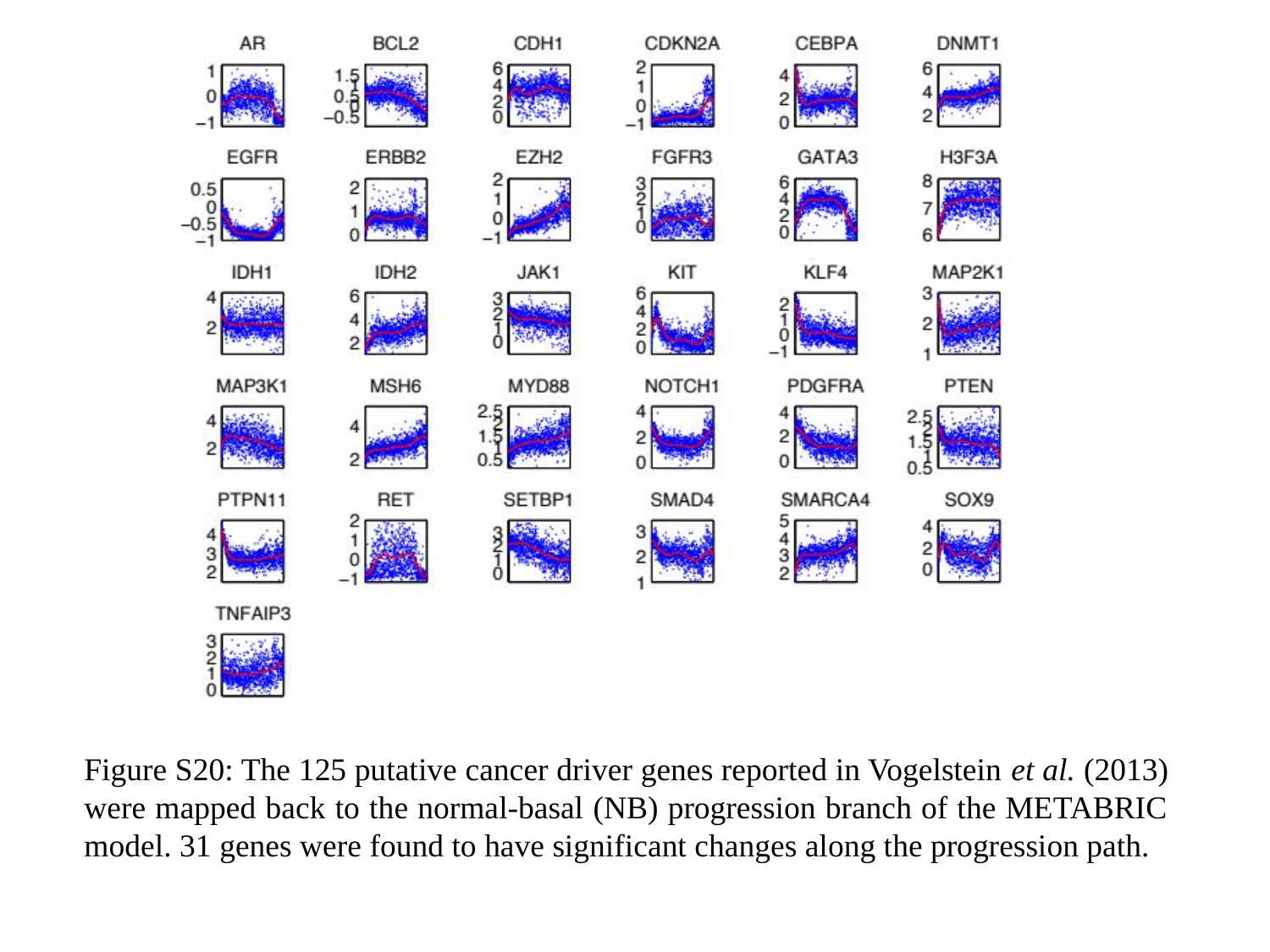

Figure S20: The 125 putative cancer driver genes reported in Vogelstein et al. (2013) were mapped back to the normal-basal (NB) progression branch of the METABRIC model. 31 genes were found to have significant changes along the progression path.
