# Supplementary material for: Cancer progression modeling using static sample data
Source: Genome Biol. 2014 Aug 26;15(8):440. doi: 10.1186/s13059-014-0440-0 (PMC4196119; doi:10.1186/s13059-014-0440-0)
Supplement: Additional file 2 — Supplementary data. Detailed mathematical derivations of the proposed methods and simulation studies. It also contains Figures S1 to S11. [file 13059_2014_440_MOESM2_ESM.pdf]

Breast Cancer Progression Modeling Using Static Sample Data (Supplementary Data)

Yijun Sun, Jin Yao, Norma J. Nowak, Steve Goodison

1 Identifying Cancer Progression Related Genes

Mathematically, a cancer progression trajectory can be viewed as a complex manifold with a branching structure embedded in a high-dimensional genomic space. Since only a small fraction of genes may involve in tumor growth and spread, the first step is to identify cancer progression related genes supporting a complex manifold. This problem has been extensively studied in both the statistics and oncology communities in different contexts. One of the most commonly used approaches is correlation analysis. A gene with its expression levels highly correlated with patient survival time is likely to play a role in cancer development. However, correlation analysis can only find genes with linear dependency with survival time. Moreover, by analyzing one gene at a time, correlation analysis ignores possible interactions among genes. In molecular classification, a commonly used approach is to first partition patients into a bad or good prognostic group at a predefined end point (usually 5-years survival or time to metastasis) and then perform feature selection for classification analysis [1]. A major drawback of this approach is that patients with survival time slightly larger or smaller than the end point will be put into two different groups. In order to explore the magnitude information of response variables, we propose to address the problem of selecting cancer progression related genes within the regression framework. The idea is not new, and has been widely used in the statistics and oncology community. There are a number of excellent algorithms exemplified by Lasso [2] and its variants [3, 4] that operate under a linear-model assumption. However, the dependency between gene expression changes and disease progression is unlikely to be linear. Recent results have shown that cancer progression processes can be complicated and can be represented by a phylogenetic tree-like structure [5].

We develop a new feature-selection algorithm for nonlinear regression. The basic idea is to decompose a nonlinear regression problem into a set of linear classification problems and learn the feature relevance within a large-margin framework. It is built logically based on our previous work on feature selection for high-dimensional classification problems [6]. We first develop a new approach to estimating sample responses and prediction errors. We then use a feature weighting strategy to find a feature subspace where an error function is minimized. We formulate it as an optimization problem with a well-defined objective function within the SVM framework, and solve it using an iterative approach. In

each iteration, a gradient descent based approach is derived to efficiently find a solution. The algorithm can be easily implemented and is computationally very efficient. We demonstrate the effectiveness of the algorithm by applying it to a set of synthetic datasets and a comprehensive breast cancer dataset and comparing performance with several state-of-the-art methods.

1.1 Algorithm

Suppose that we have a dataset $\mathcal{D} = \{(\mathbf{x}_n, y_n)\}_{n=1}^{N}$, where $\mathbf{x}_n \in \mathcal{R}^J$ is the n-th sample, y_n is its corresponding response and $J \gg N$. We aim at finding a feature subset so that the response of unseen samples can be optimally predicted based on some criteria. Therefore, the essence is to design a criterion to quantify prediction accuracy that can be conveniently optimized by some optimization techniques. To this end, we digress slightly and consider the nonlinear regression problem for the moment. Given a sample $\mathbf{x}$, a straightforward approach is to find a sample $\mathbf{x}^*$ in $\mathcal{D}$ that is closest to $\mathbf{x}$ and assign the response of $\mathbf{x}^*$ to $\mathbf{x}$:

$$\mathbf{x}^* = \arg\min_{\mathbf{x}_n \in \mathcal{D}} d(\mathbf{x}, \mathbf{x}_n), \quad \text{and} \quad \hat{y}(\mathbf{x}) = y(\mathbf{x}^*) \ , \tag{1}$$

where $d(\mathbf{x}, \mathbf{x}_n)$ is a distance function measuring the similarity between two samples. A general version is to use the Nadaraya-Watson method to estimate the response of $\mathbf{x}$ as:

$$\hat{y}(\mathbf{x}) = \sum_{n=1}^{N} K(\mathbf{x}, \mathbf{x}_n) y_n / \sum_{n=1}^{N} K(\mathbf{x}, \mathbf{x}_n) \ ,$$

where $K(\cdot)$ is a kernel function. A natural idea then is to find a weighted subspace parameterized by a non-negative weight vector $\mathbf{w}$ so that the objective function $\sum_{n=1}^{N} f(y_n, \hat{y}(\mathbf{x}_n|\mathbf{w}))$ is minimized, where $f(y_n, \hat{y})$ is a cost function, which can be $|y_n - \hat{y}|$ or $(y_n - \hat{y})^2$, and

$$\hat{y}(\mathbf{x}_n|\mathbf{w}) = \frac{\sum_{i=1, i\neq n}^{N} K(\mathbf{x}_n, \mathbf{x}_i|\mathbf{w}) y_i}{\sum_{i=1, i\neq n}^{N} K(\mathbf{x}_n, \mathbf{x}_i|\mathbf{w})} \ . \tag{2}$$

This is the formulation proposed by [7] and is optimized by using a gradient descent method. One major issue with the above formulation is that there is no guarantee that an optimal solution can be found due to the presence of local minima.

We propose a new approach to estimating sample responses and prediction errors. Without loss of generality, we assume that $y_i \geq y_j$ if $i > j$. For every y_n, $2 \leq n \leq N$, we compute $s = (y_{n-1} + y_n)/2$ and divide the dataset $\mathcal{D}$ into two subsets $\mathcal{D}_1 = \{\mathbf{x}_i | y_i < s, 1 \leq i \leq N\}$ and $\mathcal{D}_2 = \{\mathbf{x}_i | y_i > s, 1 \leq i \leq N\}$. Given a sample $\mathbf{x}$, we compute two distances: $d_1(y_n) = \min_{\mathbf{z} \in \mathcal{D}_1} d(\mathbf{x}, \mathbf{z})$, and $d_2(y_n) = \min_{\mathbf{z} \in \mathcal{D}_2} d(\mathbf{x}, \mathbf{z})$. We determine that the response of $\mathbf{x}$ is larger than or equal to y_n if $d_1 > d_2$, and smaller than y_n otherwise. The above described test is repeated starting from y_2 until we find a y_n so that $d_1 \leq d_2$. Then, the response of $\mathbf{x}$ is estimated to be y_{n-1}. Let $\triangle d(y_n) = d_1(y_n) - d_2(y_n)$. It can be proved that $\triangle d(y_n)$ is a monotonically decreasing function of y_n. This means that once we find y_n there is no need to perform additional tests. Also, it is easy to prove that the response y_{n-1} estimated in the above

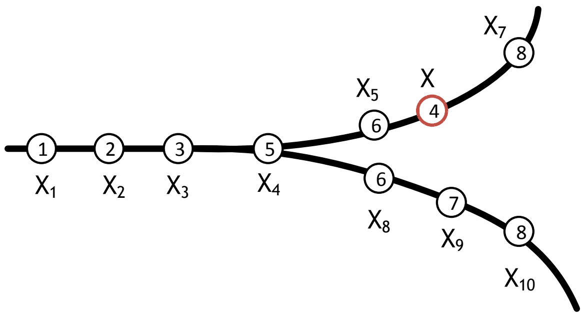

Figure S1: Toy example. The number in each circle is the response of the corresponding sample.

test is equal to $\hat{y}$ estimated in (1). However, we will shortly see that the approach we use to estimate sample responses enables us to circumvent the local minimum problem.

Let y be the true response of $\mathbf{x}$, and define $\rho(\mathbf{x}|y_n) = \triangle d(y_n)\text{sign}(y - y_n)$. We define the prediction error as the number of operations one needs to perform in order to move $\mathbf{x}$ from the predicted response to the correct one. We illustrate this using a toy example presented in Fig. S1. If we are only allowed to move $\mathbf{x}$ one position at a time, it requires at least two operations to shift $\mathbf{x}$ to the right position. Mathematically, the prediction error can be computed as $\epsilon(\mathbf{x}) = \sum_{n=1}^{N} \text{I}(\rho(\mathbf{x}|y_n) < 0)$, where $\text{I}(x < 0)$ is an indicator function that takes the value of 1 if $x < 0$ and 0 otherwise. The above definition can be interpreted within the classification framework: if we successively divide a dataset into two subsets and use the one-nearest-neighbor classifier to classify $\mathbf{x}$ into one of the two groups, $\epsilon(\mathbf{x})$ equals to the number of times when $\mathbf{x}$ is misclassified, and $\rho(\mathbf{x}|y_n)$ can be interpreted as a margin of $\mathbf{x}$. This presents a close connection between regression and classification problems.

Once we define a prediction error function, we proceed to find a weighted feature subspace where the overall prediction error is minimized:

$$\min_{\mathbf{w} \geq 0} \sum_{n=1}^{N} \sum_{\{i=1, i \neq n\}}^{N} \text{I}(\rho(\mathbf{x}_n|y_i, \mathbf{w}) < 0) \ . \tag{3}$$

Since in the inner summation $\mathbf{x}_n$ is held out as a test sample, the above objective function can be interpreted as a leave-one-out cross-validation error. For the purpose of this paper, we use the block distance to measure the similarity between two samples, which is also used in the RELIEF [8] and LOGO algorithms [6]. Let $\text{NN}(\mathcal{D}_1)$ and $\text{NN}(\mathcal{D}_2)$ be the nearest neighbors of $\mathbf{x}_n$ in $\mathcal{D}_1$ and $\mathcal{D}_2$, respectively. Then, $\rho(\mathbf{x}_n|y_i, \mathbf{w})$ can be computed as a linear function of $\mathbf{w}$:

$$\rho(\mathbf{x}_n|y_i, \mathbf{w}) = \mathbf{w}^T \Big(|\mathbf{x}_n - \text{NN}(\mathcal{D}_1)| - |\mathbf{x}_n - \text{NN}(\mathcal{D}_2)| \Big) \text{sign}(y_n - y_i) \triangleq \mathbf{w}^T \mathbf{z}_n(i) \ ,$$

where $|\cdot|$ is an element-wise absolute operator. The problem (3) can now be simplified as:

$$\min_{\mathbf{w}\geq 0} \sum_{n=1}^{N} \sum_{\{i=1,i\neq n\}}^{N} \mathrm{I}(\mathbf{w}^T\mathbf{z}_n(i) < 0) \ . \tag{4}$$

Note that the indicator function is non-differentiable and non-convex. A commonly used practice to address the issue is to minimize the upper bound of a cost function. We use the hinge loss, which leads to a SVM formulation of feature selection for nonlinear regression:

$$\min_{\mathbf{w}} \sum_{n=1}^{N} \sum_{\{i=1,i\neq n\}}^{N} \max\left(0, 1-\mathbf{w}^T\mathbf{z}_n(i)\right) , \ \text{subject to } \|\mathbf{w}\|_1 \leq \lambda, \mathbf{w} \geq 0, \tag{5}$$

where we impose an ℓ_1 penalty on $\mathbf{w}$ in order to obtain a sparse solution, and λ is a regularization parameter controlling the sparseness of a solution.

There are a number of algorithms that can be used to solve the ℓ_1-SVM problem (see, for example, [9]). We demonstrate here that ℓ_1-SVM can be easily solved in its primal domain by using gradient descent techniques. Since the hinge loss is a non-differentiable function, we thus replace it by the Huber loss defined as

$$H(\rho) = \begin{cases} 0 & \rho > 1+h \ , \\ \dfrac{(1+h-\rho)^2}{4h} & 1-h \leq \rho \leq 1+h \ , \\ 1-\rho & \rho < 1-h \ , \end{cases}$$

where h is a tunable parameter. If h is sufficiently small, SVM using the Huber loss yields the same solution as that with the hinge loss [10].

The problem (5) is a constrained convex optimization problem. In order to use gradient descent techniques, traditional methods apply projection or barrier functions to prevent solutions from falling outside feasible regions. In this paper, we use a different approach where we convert the constrained problem into an unconstrained one by setting $w_j = v_j^2$ for $1 \leq j \leq J$. Then, the problem (5) with the hinge loss replaced by the Huber loss can be re-written as

$$\min_{\mathbf{v}} L(\mathbf{v}) = \sum_{n=1}^{N} \sum_{\{i=1,i\neq n\}}^{N} H\left(\sum_{j=1}^{J} v_j^2 z_n^j(i)\right) + \alpha \sum_{j=1}^{J} v_j^2 \ , \tag{6}$$

where α is a Lagrange multiplier. Taking the derivatives of L with respect to $\mathbf{v}$ yields

$$dL/d\mathbf{v} = 2\left(\sum_{n=1}^{N} \sum_{\{i=1,i\neq n\}}^{N} \frac{dH}{d\rho}\mathbf{z}_n(i) + \alpha\mathbf{1}\right) \odot \mathbf{v},$$

where $\mathbf{1}$ is an all-one vector and $\odot$ is the Hadamard operator. Thus, the problem (5) can be solved by using gradient descent with the following updating rule:

$$\mathbf{v}^{(k)} = \mathbf{v}^{(k-1)} - \eta\frac{dL}{d\mathbf{v}}|_{\mathbf{v}=\mathbf{v}^{(k-1)}} = \left((1-2\eta\alpha)\,\mathbf{1} - 2\eta\sum_{n=1}^{N} \sum_{\{i=1,i\neq n\}}^{N} \frac{dH}{d\rho}\mathbf{z}_n(i)\right) \odot \mathbf{v}^{(k-1)}, \tag{7}$$

where $\mathbf{v}^{(k)}$ is the solution obtained at the k-th iteration, and η is a learning rate that can be determined through a line search. Note that the objective function of (6) is no longer convex, and a gradient descent method may find a local minimizer or a saddle point. However, (6) is quasi-convex for $\mathbf{v} \geq 0$, and it can be proved that if the initial point $v_j^{(0)} \neq 0$ for $1 \leq j \leq J$, the solution obtained when the gradient vanishes is a global minimizer [11].

There are two issues associated with the above formulation. The first issue is that although local learning allows us to model complex local data structures, the nearest neighbor of a given sample is unknown before learning. In the presence of many thousands of irrelevant features, which is the case in this study, the nearest neighbors defined in the original space can be completely different from those defined in a weighted space. One possible way to address this issue, proposed by [6], is to use a probabilistic model where the nearest neighbor of a given sample is treated as hidden variables. Following the principles of the expectation-maximization algorithm [12], we estimate $\rho(\mathbf{x}_n|y_i, \mathbf{w})$ by taking expectation via averaging out hidden variables:

$$\begin{aligned}\bar{\rho}(\mathbf{x}_n|y_i, \mathbf{w}) &= \mathbb{E}[\rho(\mathbf{x}_n|y_i, \mathbf{w})] = \mathbf{w}^T \Big(\mathbb{E}_{j\sim\mathcal{M}_1}[|\mathbf{x}_n - \mathbf{x}_j|] - \mathbb{E}_{j\sim\mathcal{M}_2}[|\mathbf{x}_n - \mathbf{x}_j|]\Big)\text{sign}(y_n - y_i) \\ &= \mathbf{w}^T \left(\sum_{j\in\mathcal{M}_1} Q(j|n, \mathbf{w})|\mathbf{x}_n - \mathbf{x}_j| - \sum_{j\in\mathcal{M}_2} P(j|n, \mathbf{w})|\mathbf{x}_n - \mathbf{x}_j|\right) \text{sign}(y_n - y_i) \triangleq \mathbf{w}^T\bar{\mathbf{z}}_n(i)\,,\end{aligned}$$

where $\mathcal{M}_1 = \{j : \mathbf{x}_j \in \mathcal{D}_1\}$, $\mathcal{M}_2 = \{j : \mathbf{x}_j \in \mathcal{D}_2\}$, $\mathbb{E}_{j\sim\mathcal{M}_1}$ is expectation taken with respect to $\mathcal{M}_1$, and $Q(j|n, \mathbf{w})$ and $P(j|n, \mathbf{w})$ are the probabilities of $\mathbf{x}_j$ being the nearest neighbors of $\mathbf{x}_n$ in $\mathcal{D}_1$ and $\mathcal{D}_2$ with respect to $\mathbf{w}$, respectively. The probability $Q(j|n, \mathbf{w})$ can be estimated through a kernel method

$$Q(j|n, \mathbf{w}) = \frac{K(\mathbf{x}_j, \mathbf{x}_n|\mathbf{w})}{\sum_{m\in\mathcal{M}_1} K(\mathbf{x}_m, \mathbf{x}_n|\mathbf{w})}\,,$$

where $K(d)$ is a kernel function. $P(j|n, \mathbf{w})$ can be computed similarly. In this paper, we use the exponential kernel given by $K(d) = \exp(-d/\sigma)$, where kernel width σ determines the resolution at which data is analyzed.

The second issue is that $\bar{\mathbf{z}}_n$ implicitly depends on $\mathbf{w}$ through $P(j|n, \mathbf{w})$ and $Q(j|n, \mathbf{w})$. We use a fixed-point recursive method to solve for $\mathbf{w}$. First, we make a guess on a weight vector $\mathbf{w}$ and compute the pairwise distances to estimate $P(j|n, \mathbf{w})$, $Q(j|n, \mathbf{w})$ and $\bar{\mathbf{z}}$, and then update the feature weight vector by solving the problem (6). The iterations are carried out until convergence.

It can be proved by using the fixed-point theory that if the kernel width is properly selected, the algorithm converges to a unique solution for any nonnegative initial feature weights [6]. This property has an important consequence: even if the initial feature weights were wrongly selected and the algorithm started computing erroneous nearest neighbors for each sample, the algorithm will eventually converge to the same solution obtained as if one had *perfect* prior knowledge on which features are useful since it is a fixed-point method.

1.2 Simulation Study

Before we apply the new algorithm to cancer transcriptome data, we present some numerical experiments to compare our method with four state-of-the-art methods, namely, RGS [7], sparse additive model (SpAM) [13], HSIC Lasso [14], and hierarchical multiple kernel learning (HMKL) [15].

Four synthetic datasets are used in the simulation study. The first dataset is generated from an additive model

$$Y = -2\sin(2X_1) + X_2^2 + X_3 + \exp(-X_4) + \mathcal{N}(0,1),$$

where $\{X_j\}_{j=1}^4 \sim \mathcal{N}(0,1)$ are independently drawn from a Gaussian distribution with zero mean and unit variance. The second dataset is generated from a non-additive model

$$Y = X_1 \exp(2X_2) + X_3^2 + \mathcal{N}(0,1),$$

where $\{X_j\}_{j=1}^3 \sim \mathcal{N}(0,1)$. The first two datasets are also used in [14] to test HSIC Lasso and SpAM. The third dataset is generated from a sine model representing the case where data has a weak linear dependency with responses:

$$Y = \sin(2\pi X) + \mathcal{N}(0, 0.1),$$

where $X \sim \mathcal{U}(0,4)$ is independently drawn from a uniform distribution $[0,4]$. The fourth dataset is generated from a spiral model

$$X_1 = Y\sin(Y) + \mathcal{N}(0,1),\ X_2 = Y\cos(Y) + \mathcal{N}(0,1),$$

where $Y \sim \mathcal{U}(0,20)$. For each dataset, the set of original features is augmented by 1000 irrelevant features, independently sampled from $\mathcal{N}(0,1)$. Our goal is to recover true signals that are completely buried in random noise.

The codes of RGS, SpAM, HSIC Lasso, HMKL are downloaded from the authors' websites, and the default parameters are used. For HSIC Lasso and HMKL, one needs to specify a regularization parameter. We run the two algorithms multiple times for each dataset using different parameters ($[1, 10, 20, \cdots, 100]$ for HSIC Lasso and $[10, 10^0, \cdots, 10^{-7}]$ for HMKL), and report the best result. The regularization parameter of SpAM is estimated by using the C_p statistics given in [13]. For our method, we simply set the kernel width $\sigma = 1$ and the regularization parameter $\lambda = 1$. Before learning, we scale the values of each feature into $[0,1]$ so that they are comparable, and no other preprocessing is performed. We apply the five methods to each dataset, rank the resulting feature weights in a descending order. If there are d useful features, the probability of correct recovery is defined as the fraction of the useful features detected in the top d features. This criterion is also used in [14, 13]. The experiment is repeated 50 times. HMKL is computationally very expensive, and it would take HMKL two months to finish the entire analysis. We thus run the algorithm only 10 times using a computer cluster.

Fig. S2 reports the probabilities of correct recovery of the five algorithms as a function of the number of samples ranging from 100 to 300, averaged over 50 runs (10 runs for HMKL). RGS performs poorly

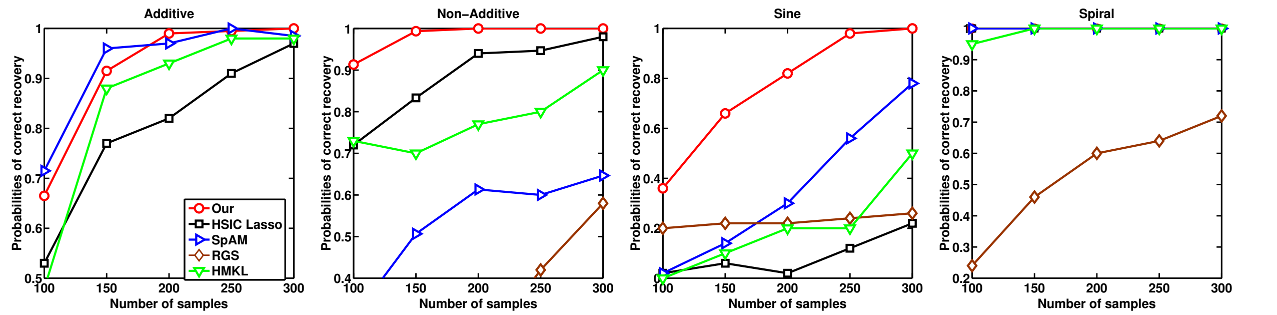

Figure S2: Probabilities of correct recovery of five algorithms applied to four datasets with 1000 irrelevant features. The probabilities of correct recovery of RGS are close to zero for the additive data.

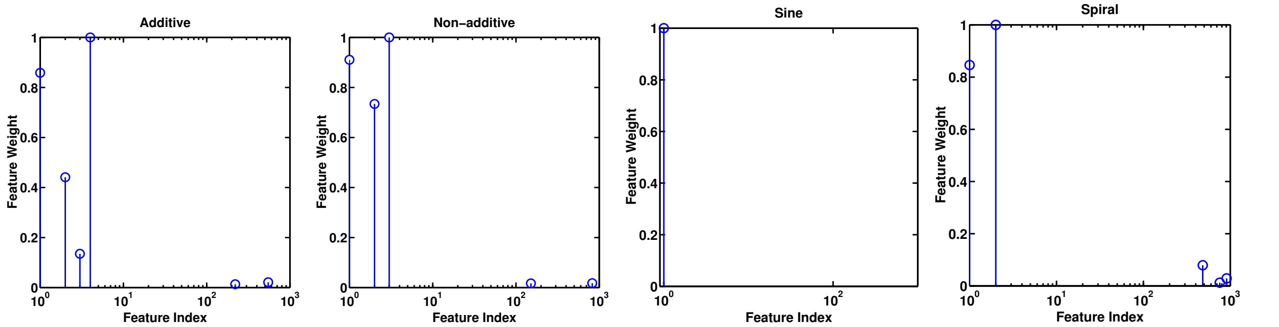

Figure S3: Feature weights generated by our algorithm applied to the four datasets with 200 samples each containing 1000 irrelevant features. Our algorithm removes nearly all irrelevant features.

on all four datasets. One possible reason is that with the increase of data dimensionality, the chance of RGS being trapped by local minima is increased exponentially. This suggests that RGS is not suitable for high-dimensional data. The performance of HSIC Lasso on the additive and non-additive data is similar to that reported in [14]. HSIC Lasso yields a perfect result on the complex spiral data, but fails on the sine data. SpAM performs extremely well on the additive dataset. This is not surprising since it is designed specifically for handling data generated by an additive model. However, SpAM performs poorly on both non-additive and sine data. This is clearly undesirable for this study since genes do interact with each other. In contrast, our method does not make any model assumption. It performs perfectly on the spiral data and comparably with SpAM on the additive data, and outperforms the four competing methods by a large margin in the other two datasets. This experiment demonstrates the versatility of our method. Fig. S3 plots the feature weights generated by our algorithm for the four datasets with 200 samples. Our algorithm detects all useful features and removes nearly all irrelevant ones.

We perform some additional studies to demonstrate various properties of the algorithm. Fig. S4

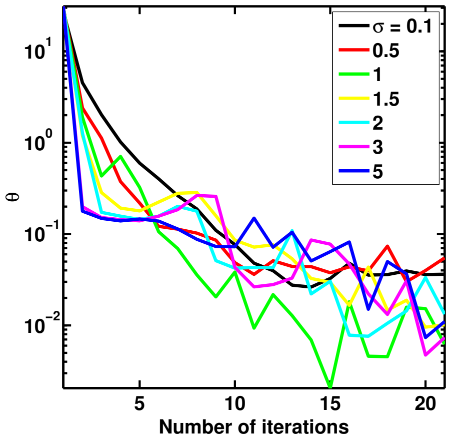

Figure S4: Convergence analysis of our algorithm performed on the non-additive data with 1000 irrelevant features, using $\lambda = 1$ and different kernel widths ranging from 0.1 to 5, where $\theta = \|\mathbf{w}^{(t)} - \mathbf{w}^{(t-1)}\|_2$.

reports the results of a convergence analysis of our algorithm applied to the non-additive data with 1000 irrelevant features, using $\lambda = 1$ and different kernel widths ranging from 0.1 to 5. We observe that the algorithm converges for a wide range of kernel width values, suggesting that our algorithm generally has no divergence issue.

The kernel width and regularization parameter are two input parameters of the algorithm. In Fig. S5, we plot the feature weights learned on the non-additive data with 1000 irrelevant features by using different kernel widths and regularization parameters. The algorithm performs well over a wide range of parameter values, which suggests that the performance of our algorithm is largely insensitive to the choice of the parameters.

1.3 Breast Cancer Dataset

We next apply our method to the METABRIC breast cancer dataset [16]. It contains the expression levels of 25,160 genes and gene copy number of 30,566 genes in 1,992 tumor samples with disease survival times ranging from 0 to 25 years. Among them, 1,486 samples are censored. In order to minimize the compounding factor of censoring, we remove samples with less than ten-year follow-up, resulting in a total of 1,147 fully annotated samples for inclusion in our analysis. We do not use RGS in the experiment as RGS does not perform well for high-dimensional data as shown in the previous simulation study. HMKL is also not suitable for this study due to its high computational complexity. In order to justify the use of a nonlinear model, we compare our method with Lasso [2]. The kernel width of our method is set to be 1, and the regularization parameters of our method, Lasso and SpAM are estimated through ten-fold cross validation. In order to make all features comparable and remove outlier data, we apply robust linear scaling [17] to each gene so that the expression quantiles 2% and 98% are set to 0 and 1, respectively. No other preprocessing is performed.

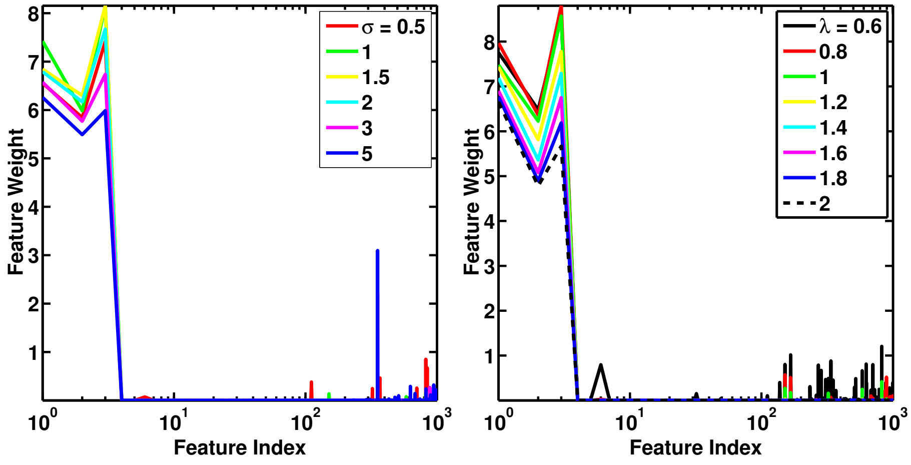

(a) (b)

Figure S5: Feature weights learned on the non-additive data with 1000 irrelevant features. (a) Using a fixed regularization parameter $\lambda = 1$ and different kernel widths $\sigma \in \{0.5, 1, 1.5, 2, 3, 5\}$. (b) Using a fixed kernel width $\sigma = 1$ and different regularization parameters $\lambda \in \{0.6, 0.8, 1, 1.2, 1.4, 1.6, 1.8, 2\}$. The performance of our algorithm is largely insensitive to the choice of the parameters.

We demonstrate the effectiveness of the proposed method in two ways. First, we compare the prediction accuracy of regression analysis performed on the features selected by four methods. To this end, we first randomly partition the dataset into two sub-datasets, one with 917 samples for training and one with the remaining 230 samples for testing. We apply each method to the training dataset to identify a list of relevant features and construct a prediction model which is then tested blindly on the test dataset. Both Lasso and SpAM can perform feature selection and prediction simultaneously. In order to use the features selected by our method and HSIC Lasso, the Nadaraya-Watson method is used. The experiment is repeated ten times. Fig. S6 presents the prediction errors of the four methods. We can see that the prediction error of our method is significantly smaller than those obtained by the three competing methods (p-value<0.05 based on Student's T-test).

We next examine whether the features selected by the four methods enable us to identify possibly nonlinear data structures consistent with those reported in the literature. Specifically, we perform spectral clustering analysis to detect genetically homogenous groups based on the profiles of the selected genes and copy number, and then compare the clustering results with breast cancer molecular subtypes. The technique of spectral clustering is detailed in the main text. Note that there are currently no widely accepted molecular subtyping methods [18, 19]. We thus compare with seven major molecular subtyping methods developed in the last decade, including SSP2003 [20], SSP2006 [21], PAM50 [22], SCMOD1 [23], SCMOD2 [24], SCMGENE [25] and IntClust [16]. We use normalized mutual information (NMI) and adjusted rand index (ARI), the two most commonly used evaluation metrics in the machine

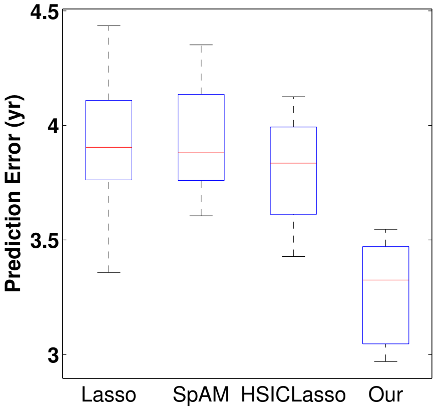

Figure S6: Prediction errors of Lasso, HSIC Lasso, SpAM and our proposed method. Prediction error is defined as the median absolute difference between predicted responses and true responses.

learning community [26], to measure the concordance of the clustering results generated by two different algorithms. Fig. S7 reports the NMI and ARI scores of the four methods. We can see that our algorithm performs significantly better (p-value<0.05 based on one-sided Wilcoxon signed rank test) than all the other three algorithms.

2 Principal Curve

We use a principal curve to formally describe the general trend of data. Mathematically, a principal curve is a smooth curve going through the center of data [27, 28]. A toy example is presented in Fig. S8. This concept is first proposed by [27] as a nonlinear generalization of the first principal component line, and requires that a curve does not have a branching structure, which is quite restrictive for real-world applications. In our study, a cancer progression path is presumably a high-dimensional manifold with multiple branches. Although a dozen of principal-curve fitting algorithms have been developed in the past two decades, there is currently no method that can be used to effectively extract a self-intersected curve in a high-dimensional space (see [29] for an excellent review). In this paper, we develop a new method to address the aforementioned issue.

2.1 Algorithm

Let $D = \{\mathbf{x}_n\}_{n=1}^{N} \in \mathcal{R}^M$ be a set of observations. We assume that each sample is generated from an unknown curve in a two-step process. First, a point $\boldsymbol{\mu}_s$ is randomly selected from the curve according to a probability density function $p(s)$, and then a data point $\mathbf{x}$ is generated from $\boldsymbol{\mu}_s$ corrupted by a Gaussian noise $\mathcal{N}(\mathbf{x}|\boldsymbol{\mu}_s, \boldsymbol{\Sigma}_s)$, where $\boldsymbol{\Sigma}_s$ is a covariance matrix and s takes a value from a set Ω. In the

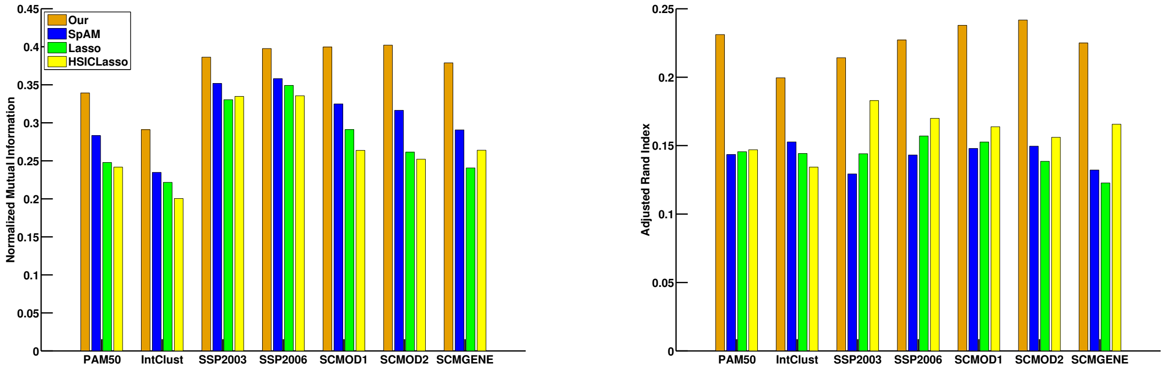

(a) NMI

(b) ARI

Figure S7: NMI and ARI scores of four methods obtained by comparing with seven existing breast cancer molecular subtyping methods.

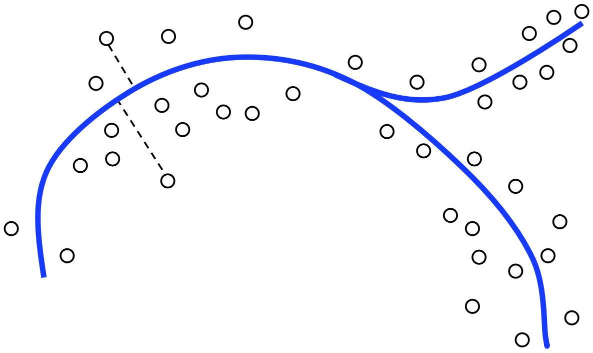

Figure S8: A principal curve is a smooth curve going through the center of a data cloud. The projection of a sample is defined as a point on the curve that is the closest to the sample.

literature, the set Ω is usually defined as a closed interval $[0, 1]$. In this paper, we do not specify any form for the set to reflect the possibility that the curve we want to learn has a branching structure. Due to the high data dimensionality ($M = 1140$), it is numerically unstable to estimate full covariance matrices, and a commonly used heuristic is to assume that $\mathbf{\Sigma}_s = \sigma^2\mathbf{I}$. Although we may lose some resolution, this simplification can lead to a numerically more stable estimation and works well for our purpose. Note that the data generalization mechanism is similar to that assumed in Gaussian mixture modeling [30]. The only difference is that in the latter case a set of discrete data points, instead of a curve, are estimated to represent an entire dataset. In this sense, principal curve fitting can be viewed as a natural extension of Gaussian mixture modeling.

Let $\boldsymbol{\theta}$ denote the parameters $\{\boldsymbol{\mu}_s, p(s)\}_{s\in\Omega}$ and σ that we try to estimate. The log-likelihood function of the data is given by

$$L(D|\boldsymbol{\theta}) = \sum_{n=1}^{N} \log \int_{s\in\Omega} \mathcal{N}(\mathbf{x}_n|\boldsymbol{\mu}_s, \sigma^2)p(s)ds \ . \tag{8}$$

The general theorem of mixture modeling suggests that with N observation, there are at most N support points [31]. Theoretically, a principal curve could be constructed by interpolating the N support points. This, however, would be difficult in a real implementation if a curve contains branching structure. Given an initial estimate of a principal curve, we discretize the continuous curve into K small segments each with a length of δ. The discrete version of the log-likelihood function thus can be written as

$$L(D|\boldsymbol{\theta}) = \sum_{n=1}^{N} \log \sum_{k=1}^{K} \mathcal{N}(\mathbf{x}_n|\boldsymbol{\mu}_k, \sigma^2)p(k) \ . \tag{9}$$

The parameters can be estimated by maximizing the log-likelihood function using the expectation-maximization (EM) algorithm [12] through an iterative process, and the estimates of $\{\boldsymbol{\mu}_s\}_{s\in\Omega}$ specify a principal curve. A detailed mathematical derivation is given in Section 2.2.

A major issue with the above formulation is that the maximum likelihood (ML) estimation is an ill-posed problem and can lead to severe over-fitting. In the literature, this is called the singularity problem [28, 30]. Specially, when σ goes to zero, $L(D|\boldsymbol{\theta})$ achieves the maximum value of infinite. In our context, it leads to a trivial solution where the resulting principal curve goes through every data point. One possible remedy for the problem is to add a regularization term to the log-likelihood function to encourage curve smoothness [28]. This strategy, however, works only for a simple case where a curve is not self-intersected since it is difficult to calculate curve smoothness for a self-intersected curve. Moreover, it is difficult to determine the regularization parameter that controls the tradeoff between data fitting and curve smoothness. It has been shown in [32] that cross-validation is not viable approach for parameter estimation as the curve fitting error always drops with the increase of curve complexity.

We address the issue by exploiting the analogy between principal curve fitting and Gaussian mixture modeling that we discuss previously. Since the estimation of σ causes the singularity problem, we treat σ as a user defined parameter and only perform the ML estimation on $\boldsymbol{\mu}_s$ and $p(s)$ for a given parameter. Now the problem becomes how to determine the value of σ. It can be shown experimentally that with

the decrease of σ, the curve complexity measured as the total curve length increases and the data fitting error decreases monotonically. In our implementation, a continuous curve is discretized into multiple equally spaced segments, and thus the curve length can be directly translated into the number of segments. The problem now becomes how to estimate the optimal number of segments, which is similar to that of estimating the number of clusters in Gaussian mixture modeling. There is a large body of work on this topic in the clustering literature [33, 34, 30]. In this study, we use the elbow method [34] for parameter estimation. Fig. S9 (top row) shows the curve fitting error measured as the mean of squared distances between data points and their corresponding closest points on a curve versus the curve length. The fitting error decreases monotonically as the curve length increases, but at a certain point the decrease flattens markedly. The elbow method uses the location of an elbow to estimate an appropriate parameter. In order to perform the estimation automatically, we fit a regression model consisting of two lines to the two arms of the elbow curve and estimate the data variance as the one that generates a curve with a length equal to that at the intersection of the two lines.

2.2 Estimating Parameters Using the EM Algorithm

We below give a detailed mathematical derivation of the maximal likelihood estimation of $\boldsymbol{\mu}_s$ and $p(s)$ using the EM algorithm for a given σ. Let $\boldsymbol{\theta}^{(t)} = \{\boldsymbol{\mu}_k^{(t)}, p^{(t)}(k)\}_{k=1}^K$ be the parameters estimated at the t-th iteration. The EM algorithm iterates the following two steps until convergence.

E-step: compute the posterior probability of $\mathbf{x}_n$ being generated from $\boldsymbol{\mu}_k^{(t)}$

$$p^{(t+1)}(k|\mathbf{x}_n) = \frac{p^{(t)}(k)\mathcal{N}(\mathbf{x}_n|\boldsymbol{\mu}_k^{(t)}, \sigma^2)}{\sum_{j=1}^{K} p^{(t)}(j)\mathcal{N}(\mathbf{x}_n|\boldsymbol{\mu}_j^{(t)}, \sigma^2)}, 1 \le n \le N, 1 \le k \le K \ . \tag{10}$$

M-step: re-estimate the parameters as

$$\boldsymbol{\mu}_k^{(t+1)} = \frac{\sum_{n=1}^{N} p^{(t+1)}(k|\mathbf{x}_n)\mathbf{x}_n}{\sum_{n=1}^{N} p^{(t+1)}(k|\mathbf{x}_n)}, 1 \le k \le K \ , \tag{11}$$

$$p^{(t+1)}(k) = \frac{1}{N}\sum_{n=1}^{N} p^{(t+1)}(k|\mathbf{x}_n), 1 \le k \le K \ . \tag{12}$$

2.3 Simulation Study

We first perform a simulation study on five synthetic datasets to demonstrate the effectiveness of the proposed method. The first four datasets are downloaded from [35], and the last one contains a tree-like structure. We apply the proposed method to each dataset by using a wide range of σ^2 values, and then

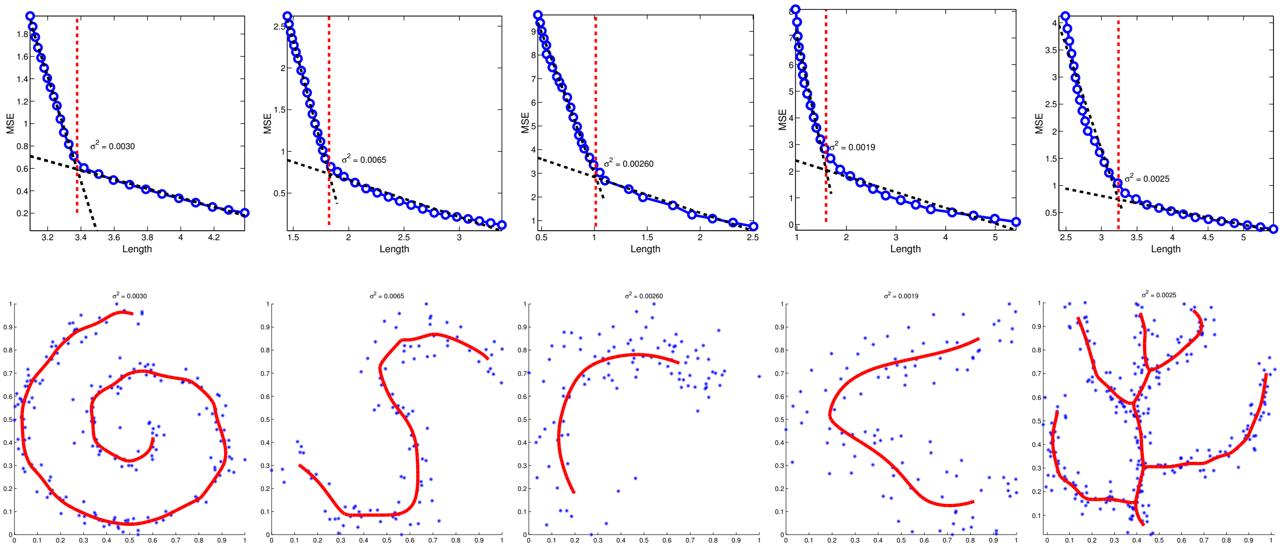

Figure S9: Simulation study performed on five synthetic datasets that demonstrates the effectiveness of the proposed method. (Top) MSE versus the curve length and the estimation of the optimal σ^2 by using the elbow method. (Bottom) The principal curves constructed by using the estimated σ^2.

compute the lengths of the constructed curves and the corresponding mean squared curve fitting errors (MSE). Fig. S9 (Top row) plots the MSE as a function of the curve length. Two straight lines are fitted to the two arms of an elbow curve to estimate the optimal σ^2 value. Fig. S9 (Bottom row) presents the principal curves constructed by using the estimated σ^2. The proposed method performs quite well in capturing the general trend of data with various structures.

We next apply the proposed method to the METABRIC breast cancer dataset. Two experiments are performed. In the first experiment, after the cancer progression related genes and copy numbers are selected by using the feature selection method described in the previous section, we perform a principal component analysis to project the data into a three-dimensional space spanning by the three leading eigenvectors, and then construct a principal curve. Fig. S10-(a) reports the MSE versus the curve length and the estimation of the optimal σ^2, and Fig. S10-(b) presents the constructed principal curve using the estimated parameter. In the second experiment, we apply the proposed method to the profiles of all selected genes and copy numbers (a total of 1140 features). The optimal σ^2 is estimated to be 0.025 and is used to construct the progression model presented in the main text.

References

[1] van't Veer, L.J., Dai, H., Van De Vijver, M.J., He, Y.D., Hart, A.A., Mao, M., Peterse, H.L., van der Kooy, K., Marton, M.J., Witteveen, A.T., *et al.*: Gene expression profiling predicts clinical

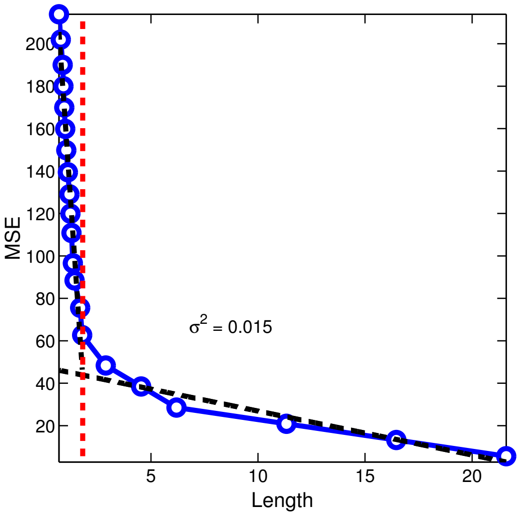

(a)

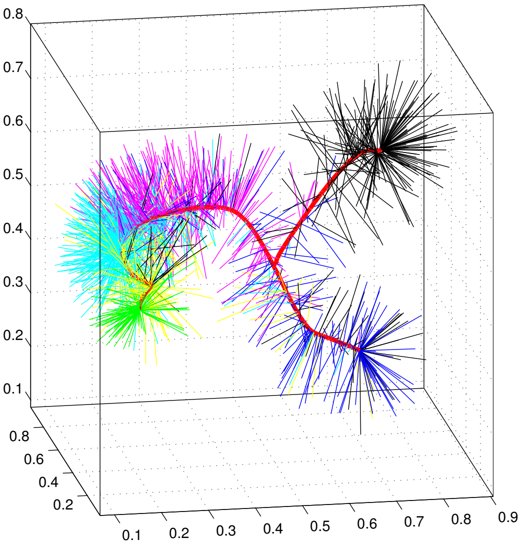

(b)

Figure S10: Principal curve fitting algorithm applied to METABRIC dataset using the three leading principal components. (a) The optimal σ^2 is estimated to be 0.015. (b) The principal curve constructed using the estimated parameter. Each sample is annotated based on its corresponding PAM50 label. Green, normal; blue, normal-like; cyan, luminal A; magenta, luminal B; yellow, HER2+; black, basal. The 3D plot can be viewed interactively in Fig. S12.

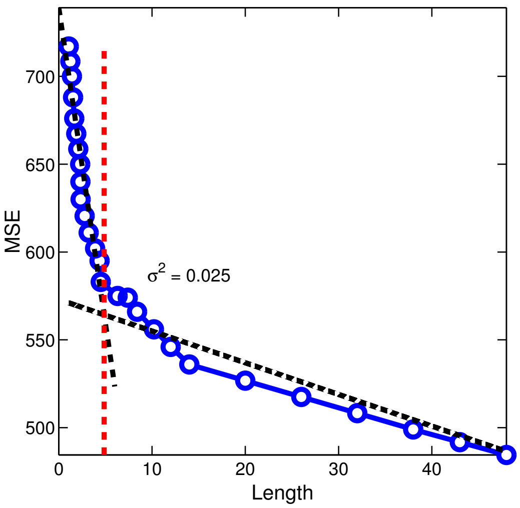

Figure S11: Parameter estimation for principal curve fitting applied to METABRIC dataset with all selected genes and copy numbers. The optimal σ^2 is estimated to be 0.025.

outcome of breast cancer. Nature **415**(6871), 530–536 (2002)

[2] Tibshirani, R.: Regression shrinkage and selection via the lasso. Journal of the Royal Statistical Society. Series B (Methodological) **58**(1), 267–288 (1996)

[3] Meier, L., Van De Geer, S., Bühlmann, P.: The group lasso for logistic regression. Journal of the Royal Statistical Society: Series B (Statistical Methodology) **70**(1), 53–71 (2008)

[4] Tibshirani, R., Saunders, M., Rosset, S., Zhu, J., Knight, K.: Sparsity and smoothness via the fused lasso. Journal of the Royal Statistical Society: Series B (Statistical Methodology) **67**(1), 91–108 (2005)

[5] Greaves, M., Maley, C.C.: Clonal evolution in cancer. Nature **481**(7381), 306–313 (2012)

[6] Sun, Y., Todorovic, S., Goodison, S.: Local-learning-based feature selection for high-dimensional data analysis. IEEE Transactions on Pattern Analysis and Machine Intelligence **32**(9), 1610–1626 (2010)

[7] Navot, A., Shpigelman, L., Tishby, N., Vaadia, E.: Nearest neighbor based feature selection for regression and its application to neural activity. In: Advances in Neural Information Processing Systems, vol. 19 (2005)

[8] Kira, K., Rendell, L.A.: A practical approach to feature selection. In: Proceedings of the Ninth International Workshop on Machine Learning, San Francisco, CA, USA, pp. 249–256 (1992)

[9] Mangasarian, O.L.: Exact 1-norm support vector machines via unconstrained convex differentiable minimization. Journal of Machine Learning Research **7**(2), 1517–1530 (2006)

[10] Chapelle, O.: Training a support vector machine in the primal. Neural Computation **19**(5), 1155–1178 (2007)

[11] Boyd, S., Vandenberghe, L.: Convex Optimization. Cambridge University Press, Cambridge (2004)

[12] Dempster, A.P., Laird, N.M., Rubin, D.B.: Maximum likelihood from incomplete data via the EM algorithm. Journal of the Royal Statistical Society. Series B (Methodological) **39**(1), 1–38 (1977)

[13] Ravikumar, P., Liu, H., Lafferty, J., Wasserman, L.: SpAM: Sparse additive models. In: Advances in Neural Information Processing Systems (2007)

[14] Yamada, M., Jitkrittum, W., Sigal, L., Xing, E.P., Sugiyama, M.: High-dimensional feature selection by feature-wise kernelized lasso. Neural Computation **26**(1), 185–207 (2014)

[15] Bach, F.: Exploring large feature spaces with hierarchical multiple kernel learning. In: Advances in Neural Information Processing Systems (2008)

[16] Curtis, C., Shah, S.P., Chin, S.F., Turashvili, G., Rueda, O.M., Dunning, M.J., Speed, D., Lynch, A.G., Samarajiwa, S., Yuan, Y., *et al.*: The genomic and transcriptomic architecture of 2,000 breast tumours reveals novel subgroups. Nature **486**(7403), 346–352 (2012)

[17] Perou, C.M., Parker, J.S., Prat, A., Ellis, M.J., Bernard, P.S.: Clinical implementation of the intrinsic subtypes of breast cancer. The Lancet Oncology **11**(8), 718–719 (2010)

[18] Mackay, A., Weigelt, B., Grigoriadis, A., Kreike, B., Natrajan, R., A'Hern, R., Tan, D.S.P., Dowsett, M., Ashworth, A., Reis-Filho, J.S.: Microarray-based class discovery for molecular classification of breast cancer: Analysis of interobserver agreement. Journal of the National Cancer Institute **103**(8), 662–673 (2011)

[19] Weigelt, B., Mackay, A., A'hern, R., Natrajan, R., Tan, D.S., Dowsett, M., Ashworth, A., Reis-Filho, J.S.: Breast cancer molecular profiling with single sample predictors: a retrospective analysis. The Lancet Oncology **11**(4), 339–349 (2010)

[20] Sorlie, T., Tibshirani, R., Parker, J., Hastie, T., Marron, J., Nobel, A., Deng, S., Johnsen, H., Pesich, R., Geisler, S., *et al.*: Repeated observation of breast tumor subtypes in independent gene expression data sets. Proceedings of the National Academy of Sciences **100**(14), 8418–8423 (2003)

[21] Hu, Z., Fan, C., Oh, D., Marron, J., He, X., Qaqish, B., Livasy, C., Carey, L., Reynolds, E., Dressler, L., Nobel, A., Parker, J., Ewend, M., Sawyer, L., Wu, J., Liu, Y., Nanda, R., Tretiakova, M., Orrico, A., Dreher, D., Palazzo, J., Perreard, L., Nelson, E., Mone, M., Hansen, H., Mullins, M., Quackenbush, J., Ellis, M., Olopade, O., Bernard, P., Perou, C.: The molecular portraits of breast tumors are conserved across microarray platforms. BMC Genomics **7**(1), 96 (2006)

[22] Parker, J.S., Mullins, M., Cheang, M.C., Leung, S., Voduc, D., Vickery, T., Davies, S., Fauron, C., He, X., Hu, Z., *et al.*: Supervised risk predictor of breast cancer based on intrinsic subtypes. Journal of Clinical Oncology **27**(8), 1160–1167 (2009)

[23] Desmedt, C., Haibe-Kains, B., Wirapati, P., Buyse, M., Larsimont, D., Bontempi, G., Delorenzi, M., Piccart, M., Sotiriou, C.: Biological processes associated with breast cancer clinical outcome depend on the molecular subtypes. Clinical Cancer Research **14**(16), 5158–5165 (2008)

[24] Wirapati, P., Sotiriou, C., Kunkel, S., Farmer, P., Pradervand, S., Haibe-Kains, B., Desmedt, C., Ignatiadis, M., Sengstag, T., Schutz, F., *et al.*: Meta-analysis of gene expression profiles in breast cancer: toward a unified understanding of breast cancer subtyping and prognosis signatures. Breast Cancer Research **10**(4), 65 (2008)

[25] Haibe-Kains, B., Desmedt, C., Loi, S., Culhane, A.C., Bontempi, G., Quackenbush, J., Sotiriou, C.: A three-gene model to robustly identify breast cancer molecular subtypes. Journal of the National Cancer Institute **104**(4), 311–325 (2012)

[26] Manning, C.D., Raghavan, P., Schütze, H.: Introduction to Information Retrieval. Cambridge University Press, Cambridge (2008)

[27] Hastie, T., Stuetzle, W.: Principal curves. Journal of the American Statistical Association **84**(406), 502–516 (1989)

[28] Tibshirani, R.: Principal curves revisited. Statistics and Computing **2**(4), 183–190 (1992)

[29] Ozertem, U., Erdogmus, D.: Locally defined principal curves and surfaces. The Journal of Machine Learning Research **12**, 1249–1286 (2011)

[30] Bishop, C.M.: Pattern Recognition and Machine Learning. Springer, New York (2006)

[31] Lindsay, B.G.: The geometry of mixture likelihoods: a general theory. The Annals of Statistics **11**(1), 86–94 (1983)

[32] Duchamp, T., Stuetzle, W.: Extremal properties of principal curves in the plane. The Annals of Statistics **24**(4), 1511–1520 (1996)

[33] Tibshirani, R., Walther, G., Hastie, T.: Estimating the number of clusters in a data set via the gap statistic. Journal of the Royal Statistical Society: Series B (Statistical Methodology) **63**(2), 411–423 (2001)

[34] Sugar, C.A.: Techniques for clustering and classification with applications to medical problems. PhD thesis, Stanford University (1998)

[35] Kégl, B., Krzyzak, A., Linder, T., Zeger, K.: Learning and design of principal curves. IEEE Transactions on Pattern Analysis and Machine Intelligence **22**(3), 281–297 (2000)
